# Biodegradable, Biocompatible, and Implantable Multifunctional Sensing Platform for Cardiac Monitoring

**DOI:** 10.1021/acssensors.3c01755

**Published:** 2024-01-03

**Authors:** Rawan Omar, Walaa Saliba, Muhammad Khatib, Youbin Zheng, Calvin Pieters, Hadas Oved, Eric Silberman, Orr Zohar, Zhipeng Hu, Viki Kloper, Yoav Y. Broza, Tal Dvir, Alon Grinberg Dana, Yan Wang, Hossam Haick

**Affiliations:** † Department of Chemical Engineering and Russell Berrie Nanotechnology Institute, Technion-Israel Institute of Technology, Haifa 3200003, Israel; ‡ Department of Chemical Engineering, Technion-Israel Institute of Technology, Haifa 320003, Israel; § Shmunis School of Biomedicine and Cancer Research, Faculty of Life Sciences, Tel Aviv University, Tel Aviv 6997801, Israel; ∥ Department Biomedical Engineering, Faculty of Engineering, Tel Aviv University, Tel Aviv 6997801, Israel; ⊥ The Chaoul Center for Nanoscale Systems, Tel Aviv University Center for Nanoscience and Nanotechnology, Tel Aviv 6997801, Israel; # Sagol Center for Regenerative Biotechnology, Tel Aviv University, Tel Aviv 6997801, Israel; ¶ Department of Chemical Engineering, Guangdong Technion-Israel Institute of Technology (GTIIT), Shantou 515063, Guangdong, China

**Keywords:** biodegradable, multifunctional, implantable sensor, health monitoring, cardiac monitoring, artificial intelligence

## Abstract

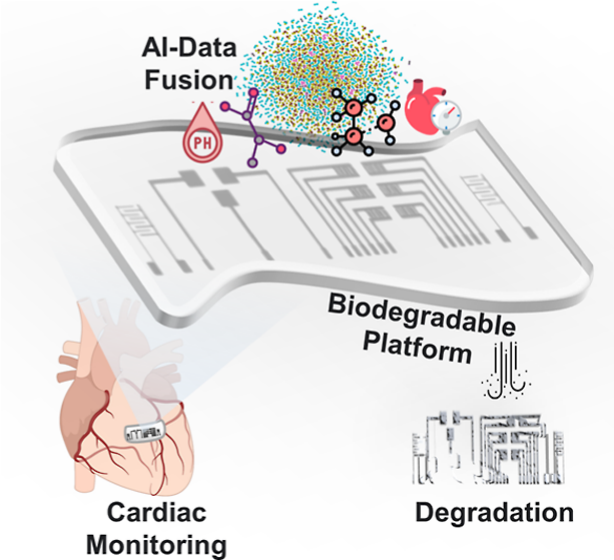

Cardiac monitoring after heart surgeries is crucial for health maintenance and detecting postoperative complications early. However, current methods like rigid implants have limitations, as they require performing second complex surgeries for removal, increasing infection and inflammation risks, thus prompting research for improved sensing monitoring technologies. Herein, we introduce a nanosensor platform that is biodegradable, biocompatible, and integrated with multifunctions, suitable for use as implants for cardiac monitoring. The device has two electrochemical biosensors for sensing lactic acid and pH as well as a pressure sensor and a chemiresistor array for detecting volatile organic compounds. Its biocompatibility with myocytes has been tested in vitro, and its biodegradability and sensing function have been proven with ex vivo experiments using a three-dimensional (3D)-printed heart model and 3D-printed cardiac tissue patches. Moreover, an artificial intelligence-based predictive model was designed to fuse sensor data for more precise health assessment, making it a suitable candidate for clinical use. This sensing platform promises impactful applications in the realm of cardiac patient care, laying the foundation for advanced life-saving developments.

Cardiovascular diseases (CVDs) are known as being a leading global cause of mortalities and morbidities, accounting for 17.9 million deaths annually, according to the World Health Organization (WHO).^1^ The impact of these diseases is significant, not just on individual patients but on healthcare systems and the economy as a whole. After heart surgeries, cardiac monitoring is a frequent practice for assessing postoperative health concerns and observing heart functioning. It is essential to monitor the heart in the hours right after surgery to sustain well-being, ward off any issues, and spot any postoperative difficulties right away.^2−4^ Unfortunately, current monitoring strategies depend on intricate devices or hard-set implants.^5,6^ Consequently, a follow-up surgery must be conducted to take out the installed device, which could possibly bring about negative health effects.^5−7^

In recent years, there has been a noticeable surge in the use of wearable and implantable devices for detecting, preventing, and treating different conditions. Fitness trackers, electrocardiography (ECG) monitors, and smartwatches, among other wearable devices, give nonstop monitoring of cardiac health and early indications of potential issues.^8−12^ Implantable devices offer an all-encompassing answer for those with serious heart conditions.^13,14^ These devices provide remarkable advantages over different external monitoring applications in healthcare and clinical settings because they can precisely keep track of vital signals within the body, thus improving patient safety and quality of life.^15^ For example, implantable cardiac monitors, such as the BioMonitor 2 device, can monitor the rhythm of the heart continuously and record the ECG for early diagnosis of arrhythmia.^16,17^ An additional example is the implantable cardiac monitor Reveal XT device for the detection of atrial fibrillation (AF).^18^ Although these devices have been proven to have good utility, the current state of implantable devices faces several challenges. One of the major challenges is that many of these rigid devices require multiple surgeries for removal. In this case, performing a second surgery to remove the implanted device is mandatory, which can lead to adverse health complications.^19^ Additionally, there are also concerns about the potential long-term health effects of having a foreign object implanted in the body.^6^ Finally, the cost of these devices remains a significant barrier for many patients, and there is a need for more affordable and accessible solutions. Additionally, the widespread use of implantable devices for the detection, prevention, and treatment of CVDs has led to a growing concern about the impact of these devices on the environment. The increase in the production of electronic waste (E-waste) due to the use of such devices is a major concern from a sustainability perspective. The Global E-waste Monitor 2020 report showed that E-waste production in 2014 was 44.4 million metric tons (Mt) and is expected to increase to 53.6 Mt by 2023. This number is projected to rise to 74.7 Mt by 2030 and could reach 78 Mt by 2050.^20^ The accumulation of E-waste presents a significant threat to the environment and human health as it contains toxic substances that can poison the aquatic environment, soil, and air. These consequences highlight the need for eco-friendly and sustainable solutions in the design and production of wearable and implantable devices.

In recent years, flexible and stretchable sensors have been developed as a more biocompatible and user-friendly alternative to rigid implantable electronics.^21,22^ However, these devices still require intervention and complex surgeries for removal, which can increase the risk of infections and inflammation. This issue highlights the need for further research and development to find sustainable solutions for the use of wearable and implantable devices in the management of CVDs.^23,24^ The pressing need for advanced and sophisticated devices to address the challenges posed by CVDs has led to increased interest in biocompatible and biodegradable devices. These devices can degrade naturally after a set period, thus avoiding the need for performing additional extraction surgeries and making them suitable for use in various fields, including environmental science,^25^ health,^26^ and food applications.^27^ In the context of clinical research and the advancements made in implantable sensors, there is a growing demand for low-cost, biodegradable, and flexible sensors that dissolve naturally. These devices are composed of bioabsorbable and biodegradable materials, do not trigger an immune response, and are safe, and nontoxic, making them ideal for clinical use as temporary medical and electronic devices that can be implanted in the body as an alternative to rigid implants.^25,28,29^ Even so, most sensors presently created, even biodegradable ones, are developed to monitor a single indicator, such as blood pressure, strain, or pulse rate. Nevertheless, for accurate, reliable, and detailed cardiac well-being analysis, multifunctional sensing of numerous biomarkers and data integration in one device is essential.^30−34^

In this study, we present an innovative method to build a multifunctional, biodegradable, and biocompatible cardiac monitor. This sensing platform can detect pressure, lactic acid, pH, and volatile organic compounds (VOCs). An artificial intelligence (AI)-generated prediction model synthesizes the readings from the sensors to generate a unique “health barcode” of the individual’s health condition. This multifunctional integration provides a comprehensive tool for effective clinical and medical assessment and cardiac monitoring as all components of the system are biocompatible and biodegradable. The proposed sensor array is flexible, easy to fabricate, and specifically calibrated to detect heart disorders through the measurement of multiple parameters. To validate the feasibility of the device, ex-vivo testing was conducted using a three-dimensional (3D)-printed silicone heart model and 3D-printed cardiac tissue patches to simulate real-life conditions. Finally, the developed platform was integrated with the internet of things (IoT) and wireless technology, such as Bluetooth or RFID, to transmit data directly to a computer or smartphone. The AI model would then process and present the data in a comprehensive manner (as depicted in Figure 1).

**Figure 1:**
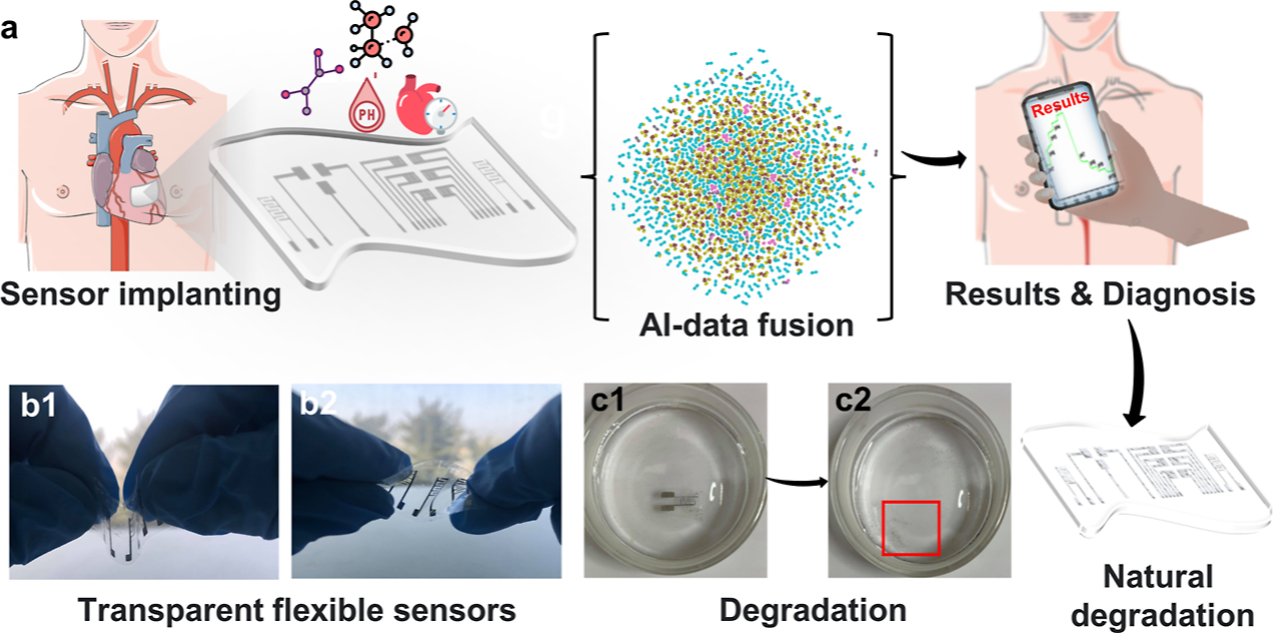
Overview of the biodegradable multiplex nanosensor platform for cardiac monitoring. (a) Concept of implantable multifunctional sensors for cardiac monitoring, including sensor implanting for detecting multiple biomarkers: pressure, lactic acid, pH and VOCs, AI-data fusion, results analysis, and degradation. (b1,b2) Flexible and bendable electrodes on polylactic acid (PLA). (c1,c2) Degradation of the sensors after a period.

## Results and Discussion

### Materials, Concept, and Sensor Fabrication

The sensor array was meticulously crafted using biocompatible and biodegradable materials, including a substrate of biodegradable poly(lactic acid) (PLA) and a biodegradable magnesium (Mg) metallic electrode as the primary conductive element (Figure S1). The array was further enhanced with the incorporation of biodegradable, bioresorbable, and biocompatible materials, such as zinc nanoparticles (Zn NPs), which were utilized to form the functional sensing layers of the various sensors. The substrates were fashioned from a transparent, flexible biodegradable PLA substrate. The electrode shape was created using a laser-cut mask, which was then attached to the PLA substrate (dimensions up to 68 mm × 17 mm). Mg was deposited onto the substrate through a thermal evaporation process, and the mask was removed to obtain the array of electrodes. The resulting electrodes exhibit high stability and conductivity (as demonstrated in Figure S2). Subsequently, a multifunctional nanosensor array, which includes pressure sensors, biosensors, and chemical sensors, was designed and fabricated on PLA in the same procedure described before, gaining an array of the multifunctional nanosensors where Mg electrodes were further used to construct the different types of sensors (Figure S1). The obtained electrodes on the PLA substrate are flexible and bendable, which broadens their range of applications in implantable soft electronics (as demonstrated in Figure 1b1,b2). The flexibility of the fabricated sensor array offers a better user experience compared with the current rigid commercial devices. This structure enables a conformal interface with tissues, and its biocompatibility reduces the side effects of implantation. Additionally, the biodegradable and biocompatible nature of the sensor array platform lowers the chances of infections, irritations, and complications compared with the commercial rigid devices used today. In addition, the methods and materials used for fabrication are simple and cost-effective, providing an advantage over currently used devices that are complicated, expensive, and made of rigid materials with complex procedures. The fact that the suggested device is biodegradable and does not require additional surgeries for removal also lowers the procedure costs of operations.^35−38^ The estimated costs of our device compared to current solutions in the market are summarized in Table S1.

### Performance of the Multifunctional Sensor Platform

#### Biodegradable Electrochemical Biosensors

**Figure 2:**
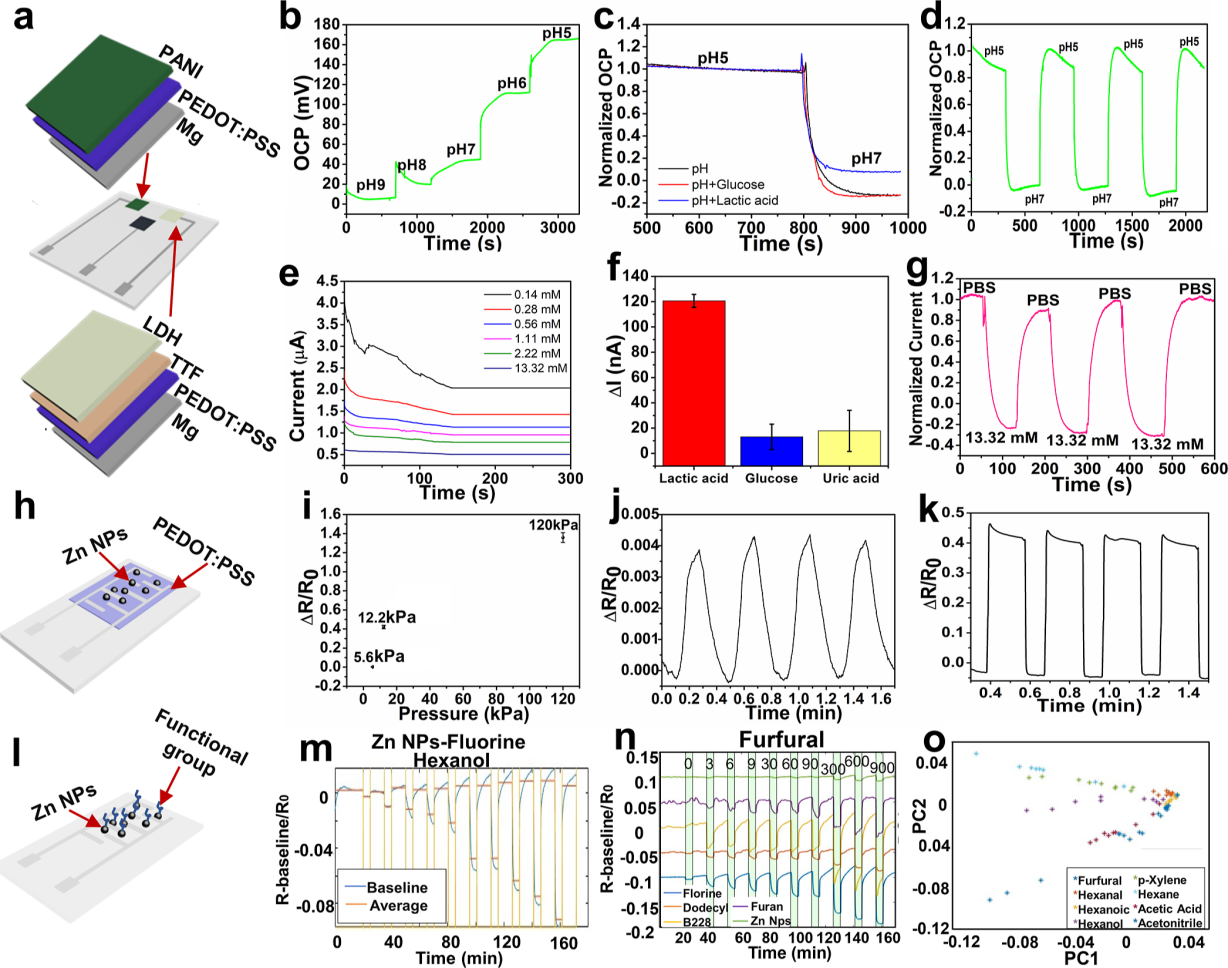
Performance of the multiplex nanosensor platform. (a) Schematic of the design of the biosensor. (b) OCP measurement and response of the pH biosensor. (c) Stability and selectivity of the pH biosensor after adding glucose and lactic acid solutions. (d) Repeatability and reversibility of the pH biosensor. (e) Electrochemical response of the lactate biosensor. (f) Selectivity of the lactate biosensor. (g) Repeatability and reversibility of the lactate biosensor. (h) Schematic of the pressure sensor design. (i) Pressure sensor response to the different pressure values. (j) Repeatability and reversibility of the sensor in 5.6 kPa. (k) Repeatability and reversibility of the sensor in 12.2 kPa. (l) Schematic of the VOC sensor design. (m) Response of the Zn NPs functionalized with fluorine to hexanol. (n) Response of the VOC sensor array to furfural. (o) PCA to differentiate and identify the different VOCs.

The development of biosensors holds immense significance in the realm of health, as they allow for precise and accurate measurement of biological and biochemical markers to assess an individual’s cardiac and overall health status.^39^ A biodegradable biosensor with dual electrochemical sensing capability has been devised (Figure 2a). One of the biosensors measure pH levels, which are crucial indicators of disease and health. In physiological conditions, a pH level higher than 7.55–7.80 or lower than 6.80 can be indicative of a fatal condition. Normal pH levels range between 7.35 and 7.45 and deviations from this range can result in metabolic alkalosis or acidosis, leading to various diseases, such as cancers, arrhythmias, cardiac, and muscle complications.^40−43^ The other biosensor is designed to detect lactate, a prominent biomarker for muscle inflammation and heart diseases. Elevated lactate levels, typically higher than 1.5−2.0 mmol/L, have been correlated with increased chances of heart attacks, heart failure, and other cardiac complications.^44−46^

The biosensors were fabricated with the main electrodes produced in the same manner as described in the previous section, with a reference electrode and a sensing electrode made of biocompatible and bioresorbable materials. To create a pH sensor, a protective layer of poly(3,4-ethylenedioxythiophene) polystyrene sulfonate (PEDOT:PSS) was added over the Mg electrode. The PEDOT:PSS complex offers outstanding biocompatibility and water solubility, making it the perfect candidate for the fabrication of biomedical devices, including implants, due to its ability to sustain cell viability.^47−49^ Then, a pH-sensitive conductive polymer, polyaniline/poly(vinyl alcohol) (PANI/PVA) composite, was sprayed to form the pH biosensor. By adding PVA, a biodegradable and water-soluble polymer, to PANI, it can be transformed into a biodegradable composite that is suitable for use in biomedical implant applications, as demonstrated in previous research studies.^50−52^ The sensor’s electrochemical response was tested using open circuit potential (OCP) with various pH solutions from 9 to 5, resulting in the PANI’s resistance changing reversibly due to protonation/deprotonation. The OCP (mV) values increased as the pH values decreased from basic to acidic (Figure 2b). A strong linear correlation was observed (*r*^2^ = 0.89) in response to pH changes with a sensitivity of 38.85 mV/pH (Figure S3). The pH sensor’s stability and selectivity were tested by changing the pH value from 5 to 7 (black curve), and the response was unchanged even after adding glucose, indicating the sensor’s stability and selectivity (red curve). Adding lactic acid slightly altered the curve by raising the OCP value, which was expected because lactic acid is acidic and causes the solution to become more acidic (a blue curve) (Figure 2c). The sensor also showed excellent repeatability and reversibility when the pH values were changed from 5 to 7 over three cycles (Figure 2d). A lactate sensor was created by first adding a protective layer of PEDOT:PSS followed by a mediating layer of tetrathiafulvalene (TTF) for increased electron transfer and then a layer of lactate dehydrogenase (LDH) enzyme embedded in chitosan and Zn NPs. The response of the sensor to different lactate concentrations was measured from 0.14 to 13.32 mM, knowing that normal lactate levels in the body are typically less than 1 mM, and higher lactate levels indicating acute CVDs. A strong linear correlation was observed (*r*^2^ = 0.97) in response to lactate changes with a sensitivity of 1.11 μA/decade (Figure S3). The sensor’s selectivity was tested against different molecules, including uric acid and glucose, and found to be significantly selective to lactic acid, with a difference of 120 nA (Figure 2f). The sensor also showed repeatable and stable behavior over three cycles (exposed each time to 13.32 mM lactate; Figure 2g).

#### Biodegradable Pressure Sensor for Physical Sensing

A pressure sensor made from a protective layer of PEDOT:PSS polymer embedded with biodegradable Zn NPs was developed (Figure 2h). This type of sensor is important in detecting physical changes, such as heartbeats, rhythm, and blood pressure, to determine if the heart is functioning normally. Additionally, the pressure sensor in the array helps to counteract external pressure applied to the entire array, allowing for accurate readings of the other sensors’ responses without external influences. The standard ratio of systolic to diastolic blood pressure is 120/80 mmHg, as determined by The National Heart, Lung, and Blood Institute.^53^ Therefore, measuring this parameter is critical in detecting abnormal blood pressure conditions. The developed pressure sensor showed effective measurement of low pressures (5.6 kPa), intermediate pressures (12.2 kPa), and high pressures (120 kPa) (Figure 2i), with excellent repeatability and reversibility (Figure 2j,k).

#### Biodegradable Chemical Sensor Array for VOC Sensing

Health monitoring and diagnosis through the detection of VOCs is being recognized as a cost-effective and informative tool for numerous illnesses,^54−56^ including cancers, tuberculosis, Alzheimer’s, and Parkinson’s diseases.^10,57,58^ Numerous VOCs are connected to metabolic changes occurring during cardiac conditions, such as lipid peroxidation, oxidative stress, and acute myocardial infarction (MI) or heart transplant rejection, including aldehydes, such as hexanal, alkanes from C_4_–C_20_ (e.g., hexane and pentane), and acetic acid.^59−66^ This paper reports the first biodegradable VOC sensor array developed for heart monitoring and CVD diagnosis. This biodegradable chemiresistor sensor was constructed with biodegradable metal nanoparticles functionalized with various functional groups. Zn NPs were selected for the array due to their unique characteristics, including biocompatibility, biodegradability, affordability, and sensitivity.^67,68^ Zn NPs tend to form an oxide layer that makes them nonconductive; one way to overcome this is by adding acetic acid to dissolve the oxide layer.^69,70^ This was followed by modification with different functional groups of thiols and chemical groups including furan, benzyl mercaptan (B228), dodecyl, fluorine, and cysteine (Cys) to produce a chemiresistor array with varying sensing abilities (Figures 2l and S4). The chemiresistor array was exposed to a range of VOCs with varying concentrations in a continuous flow, including hexanol, hexane, *p*-xylene, hexanal, furfural, acetonitrile, hexanoic acid, and acetic acid. The response of the Zn NP-fluorine sensor to hexanol at concentrations of 0, 3, 6, 9, 30, 180, 360, and 720 ppm is displayed in Figure 2m, and each chemiresistor in the array showed a unique resistance to different gases, while the limit of detection for each one of the VOCs was 3 ppm for hexanol, 40 ppm for hexane, 3 ppm for *p*-xylene, 3 ppm for hexanal, 3 ppm for furfural, 30 ppm for acetonitrile, 2 ppm for hexanoic acid, and 3 ppm for acetic acid (as shown in Figures S5 and S6). The response of the sensor array to furfural is demonstrated in Figure 2n, and the responses to the rest of the VOCs are shown in Figure S6. This differential reaction to each VOC allowed pattern recognition techniques (PCA) to differentiate and identify the different VOCs (as shown in Figure 2o). This highlights the potential of the biodegradable Zn NP-based sensor array for VOC detection.

### Biocompatibility of the Developed Sensors

**Figure 3:**
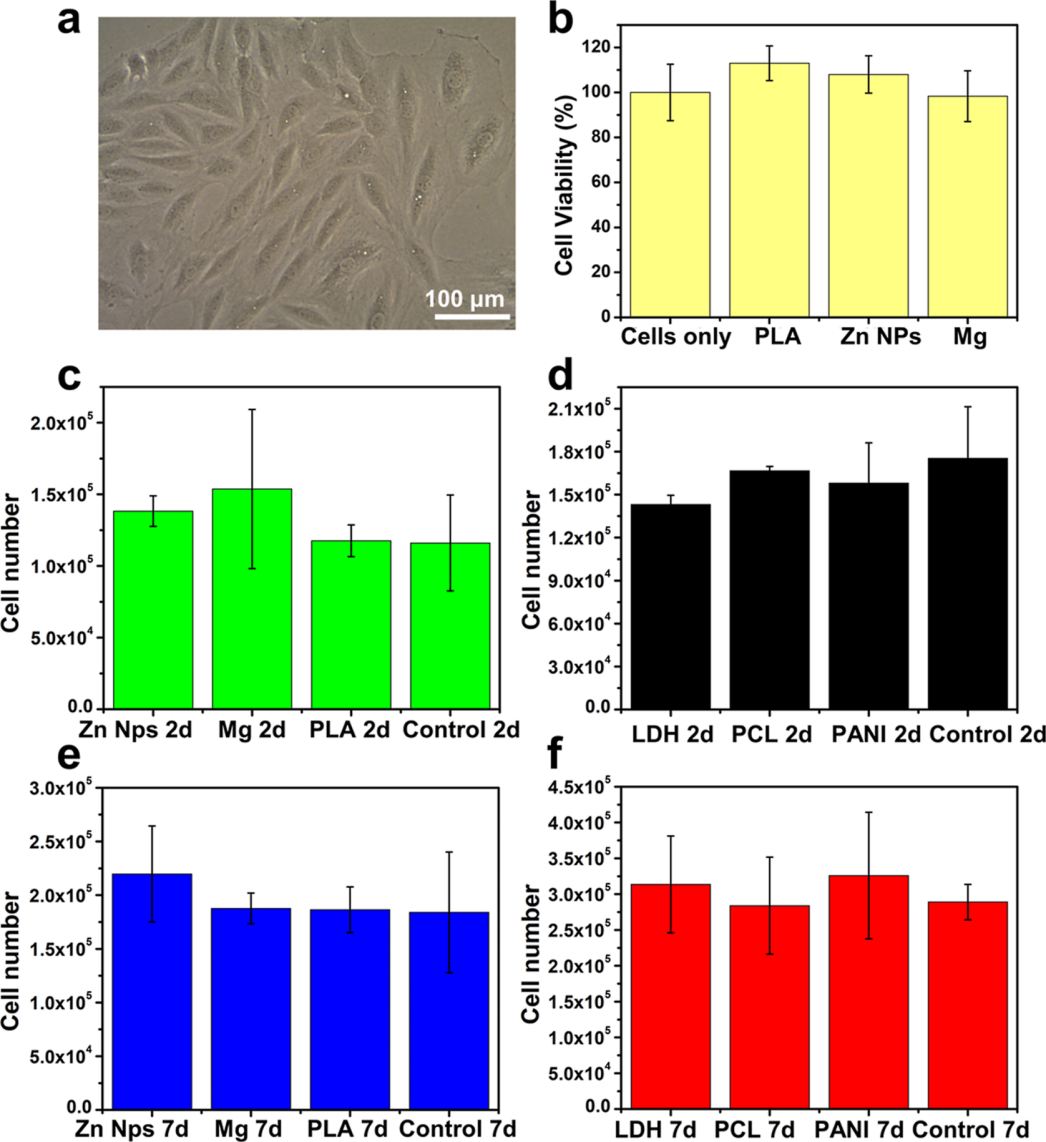
Biocompatibility and cytotoxicity tests using cardiac cells. (a) Shape and morphology of the H9c2(2–1) cardiac cells model. (b) MTT cytotoxicity results. (c) Cell number of the constituent materials after 2 days. (d) Cell number of the sensing materials after 2 days compared to the control. (e) Cell number of the constituent materials after 7 days. (f) Cell number of the sensing materials after 7 days. *n* = 3. Data are presented as mean values ± SD.

To ensure the safety and biocompatibility of the developed sensor array for use as implants; biocompatibility, cell viability, and cytotoxicity tests were conducted. The H9c2(2–1) cell line, derived from the embryonic heart tissue and representing skeletal muscle, was used for the in vitro tests (Figure 3a). The toxicity of the components was first evaluated using a 3-(4,5-dimethylthiazol-2-yl)-2,5-diphenyl-2*H*-tetrazolium bromide (MTT) assay (Figure 3b), and there was no significant change in cell viability (%) when the cells were added with PLA, Zn NPs, and Mg compared to the control cells, indicating low toxicity of the sensor array.

Biocompatibility was tested for all sensor components and their combinations, including Mg, Zn NPs, PLA, and PEDOT:PSS with PANI, PEDOT:PSS TTF, and the LDH enzyme. The cell number was counted after 2 days and 7 days (Figures 3c–f and S7), and in all cases, no significant difference was observed between the cells grown with different sensor materials and the control cells grown regularly without any addition, indicating full biocompatibility and nontoxicity. Furthermore, Mg and Zn NPs showed an encouraging effect on the proliferation of cardiac cells (Figure 3c,e), demonstrating both the biocompatibility and therapeutic benefits of using these metals in the fabrication of implantable sensors for cardiac applications. It is noteworthy that Mg and Zn have been shown to have a beneficial impact on heart cells and muscles, as it modulates oxidative stress, regulates blood pressure, and preserves the myocardial structure, thus suggesting therapeutic and rehabilitation potential for heart cells.^71−73^

### Degradation of Sensor Components

**Figure 4:**
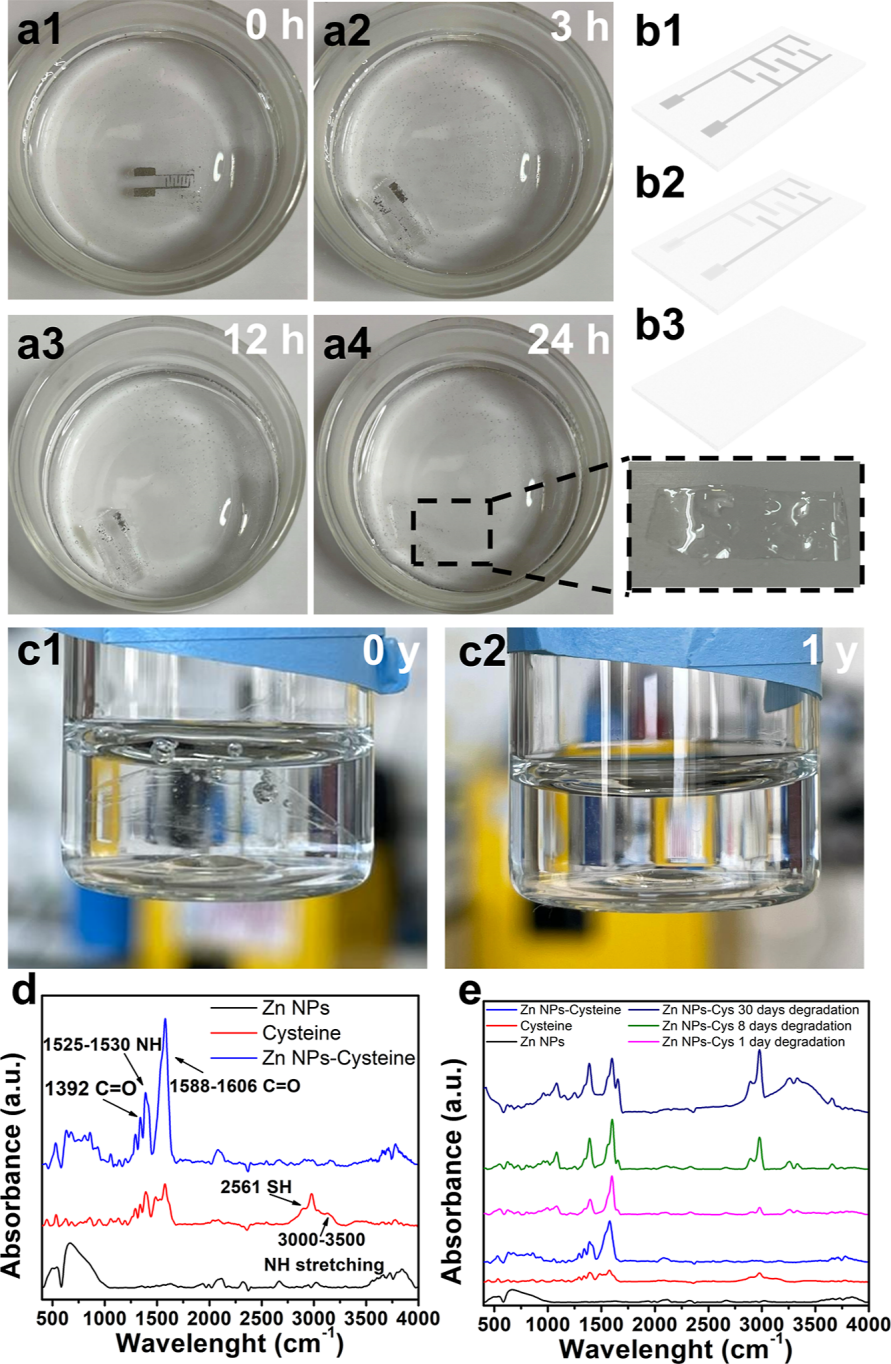
Degradation tests were carried out for the fabricated sensor array. (a1–a4) Degradation of the Mg electrode in SBF at 0, 3, 12, and 24 h accordingly. (b1–b3) Schematic of the degradation of the Mg electrode in SBF. (c1) Beginning of the degradation of PLA. (c2) Degradation of the PLA substrate in SBF after 1 year. Fourier transform infrared (FTIR) diagram of (d) the Zn NPs, a thiol, and their combination. (e) Degradation of the Zn NP-thiol in SBF over time.

The sensors are designed to degrade after a certain period in a physiological environment once their intended use has been completed. To imitate the physiological environment, a simulated body fluid (SBF) was prepared and used. The degradation of the Mg electrodes was first demonstrated and proven in the SBF. The Mg electrodes dissolved rapidly in the SBF, and complete degradation was observed within 24 h (Figure 4a1–a4,b1–b3 and Video S1). The Zn NPs degraded within 2 months (Figure S8). The degradation of the PLA took longer, taking approximately 1 year to dissolve completely in the SBF (Figure 4c1,c2).

To validate the degradation of the functionalized Zn NPs, Zn NPs-Cys was chosen as a representative functional substance and dispersed in SBF. The powder was dried at several time points, and FT-IR measurements were performed to track the degradation process over time (Figure 4d,e). The results showed that the thiol degraded over time compared to pure Zn NPs and pure Cys, as seen from the increase in the SH bond (2561 cm^–1^) and NH stretching bond (3000–3500 cm^–1^) over time, indicating that the degradation process took place. A coating made of polycaprolactone (PCL) polymer was created by using electrospinning to moderate and control the degradation time of the sensor. The membrane needed to be permeable to VOCs and highly hydrophobic to prevent the liquid from dissolving the sensor. The sensor was coated with a PCL membrane (∼0.738 mm thick) using a hot press at 60 °C, and the degradation was observed over time (Figure S9). The results demonstrated that the degradation was slower than that of the bare electrode and extended by more than 24 h. The sensor was fully coated and immersed in SBF solution and connected to a light-emitting diode (LED) to observe the illumination over time (Figure S10 and Video S2). The LED remained active over 1 week, indicating that the degradation process was moderated and slowed down. Finally, the permeability of the membrane was tested by filling a glass volumetric test tube with 4 mL of ethanol and observing evaporation over time (Figure S11). The results showed that the ethanol’s volume decreased over time, proving the permeability of the membrane.

### Ex Vivo Validations and Development of AI Prediction Model

**Figure 5:**
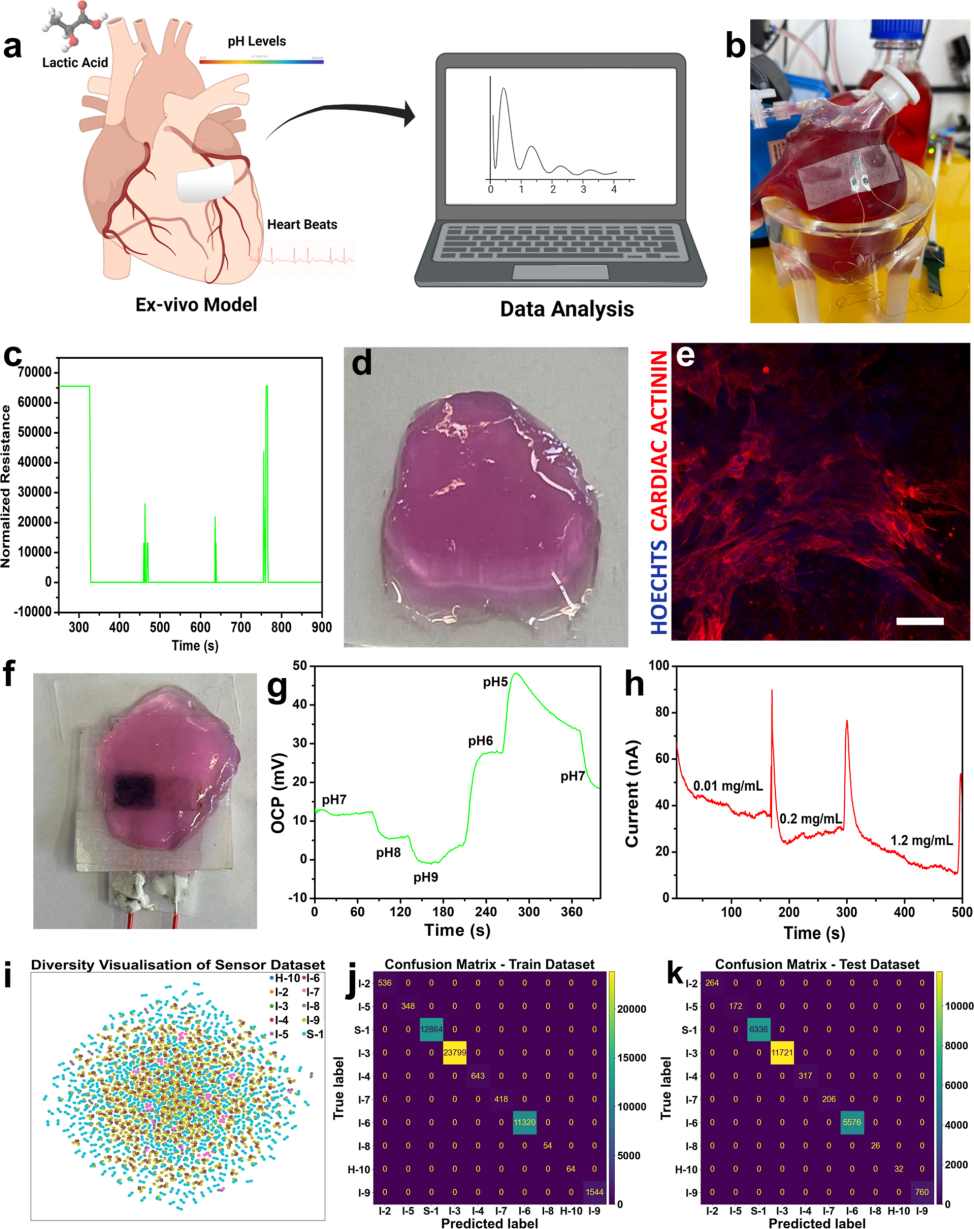
Ex vivo validation of the sensor using 3D printed models and AI-model development. (a) Overview of the ex vivo experimentations set. (b) Testing the biodegradable pressure sensor with a 3D-printed silicone heart model. (c) Pressure response with normalized resistance. (d) 3D-printed cellularized cardiac patch. (e) Confocal image showing the three-dimensional self-organization of cardiomyocytes within the printed cardiac patch (Scale bar = 50 μm). (f) Printed cardiac patch was tested with the biodegradable biosensor. (g) Response of the pH biodegradable biosensor using the cardiac patch. (h) Response of the lactate biodegradable biosensor using the cardiac patch. (i) Diversity visualization of the sensor data set. (j) Predictions on the training data set. (k) Predictions on the test data set.

The functionality of the fabricated sensors was validated through ex vivo experiments that were performed in a simulated environment similar to real-life conditions. The sensors were tested on a 3D-printed silicone heart that mimicked the beating of a real heart and on 3D-printed cardiac patches to test their performance in implantable cardiac applications (Figure 5a). To test the sensors on the beating heart, a silicone heart was 3D printed, connected to a peristaltic pump, and then fitted with a biodegradable pressure sensor. The resistance change was measured when the heart was subjected to different beating powers (Figure S12). The sensor was then connected to a Bluetooth chip, and the data was transmitted via IoT technology to the cloud and displayed on a computer (Figure 5b,c and Videos S3 and S4).

For the 3D-printed cardiac patches, live cardiac cells were used to test the sensor’s response and diffusion in the heart tissue, mimicking its use in implantable cardiac applications (Figure S13). The patches were designed with several arteries to simulate the diffusion of biomarkers in the real heart tissue (Video S5). The efficient diffusion of solvents was demonstrated by injecting a red dye (Figure S14 and Video S6). To create the patches, cells were taken from human omentum fat tissue, transformed into pluripotent stem cells (iPSCs) and then reprogrammed into cardiomyocytes. The decellularized extracellular matrix (ECM) was processed into a hydrogel, and the cells were then encapsulated within this hydrogel matrix to form a bioink that was printed into the desired shape to match the sensor. After a few days, the cells matured into beating cardiac tissue, and the contraction amplitude was calculated to validate the beating of the patch (Figures 5d,e, S14 and S15 and Videos S7 and S8).

Afterward, the diffusion of biomarkers and the response of the biosensors were measured through a 3D-printed cardiac patch tissue (as shown in Figure 5f). The patch was subjected to different pH solutions, and the electrochemical response of the pH biosensor was recorded. When the pH was changed from 7 to 5, the sensor responded as expected, showing lower OCP values in basic levels and higher values in acidic pH levels (as demonstrated in Figures 5g and S16A and Video S9). The 3D-printed cardiac patch was also tested with a biodegradable lactate biosensor, and the electrochemical response was continuously recorded as solutions of lactic acid from 0.01 to 1.2 mg/mL were injected. The sensor responded as expected to the diffusion of biomarkers in the living cardiac patch (as seen in Figures 5h and S16B and Video S10). Thus, the biosensors showed a good response in diffused 3D cardiac tissue and were biocompatible when attached to the 3D printed patch, demonstrating both good biocompatibility and good electrical performance. The ex vivo experiments provide evidence for the potential of these sensors as implantable sensors for cardiac and other health applications.

An AI prediction model was developed to perform data fusion of all sensor signals based on the experimental data sets collected, serving as a prototype for an optional method of data fusion in real-life settings. The model outputs a score from 1 to 10 based on a “health barcode” reflecting the individual’s health status, where Healthy-10 is the healthiest score, Intermediate-9 to 2 is the intermediate status, and Sick-1 is the sick status (see Supplemental Notes 1 and 2 and Tables S2–S5). The overall data set from the sensors was plotted using T-distributed Stochastic Neighbor Embedding (tSNE), showing the distribution of the final output from H-10 to S-1 from the created model (as seen in Figure 5i). Multiple models, such as a CatBoost Classifier, were trained on a 70% data split of the overall data set, and a confusion matrix of the model’s predictions for the training data was plotted (as shown in Figure 5j). The trained models were then tested against the remaining 30%. All models reported a 100% accurate prediction against the test data set (as seen in Figure 5k). While at first glance 100% accuracy is excellent, it also means that there is a possibility of “target leakage”. Target leakage occurs when the test and training data share very strong similarities and thus are arguably indistinguishable. A further investigation supported this hypothesis after finding that the models also scored 100% accurate predictions against the training data set. As it currently stands, these synthetic data can only be used for either model training or testing, not both. However, combining these data with real-world clinical data would be strongly recommended as the next step forward in developing an AI predictive model.

The advantage of the fused data over a singular sensor was emphasized by calculating the accuracy of the pH sensor, as an example. The accuracy of a singular pH sensor was compared to that of the fused sensors, showing that the accuracy would be 0.62% if the pH was used as the sole health indicator (as seen in Tables S6 and S7). The results highlight the great benefit and advantage of the data fusion of multifunctional sensors for accurate health assessment.

To further investigate the AI model, a blind data set was tested, showing the effectiveness of the model in additional data sets (detailed information is provided in Supplemental Note 3, Figures S17–S21 and Table S8). It is noteworthy that the utilization of synthetic training data, while a practical starting point, has revealed the necessity for enhancement with real-world clinical data. This integration promises a more nuanced understanding of the complex relationships within the data, potentially improving the model’s predictive capabilities. Equally crucial is addressing the skewed class distribution within the training set to mitigate any inherent bias toward more frequently occurring labels. Ensuring a balanced representation of all labels, especially those of critical importance such as “S-1”, which indicates severe sickness, is imperative. Moreover, a further granular analysis of the importance of correctly predicting specific labels is warranted. Given the potential real-world application of this AI model in clinical settings, the accuracy of certain predictions, such as the correct identification of severely sick patients, could be more consequential than the overall prediction accuracy.

With this model, physicians can diagnose more precisely and rapidly, demonstrating the potential of this new nanosensor platform for cardiac monitoring. It is biocompatible, bioresorbable, and an intelligent high-tech tool that can assist in medical decision-making and health diagnosis. Additionally, the biodegradable and biocompatible nature of the platform, coupled with its capacity to detect various parameters and employ AI in data-fusion, enhances its utility in additional health assessment applications. The platform can be calibrated for additional diseases, including those related to the digestive and nervous systems and various types of cancers. The biodegradability feature extends its applicability to additional industries, such as agriculture, leveraging the eco-friendly materials that degrade without environmental harm. Consequently, this universal platform holds promise in diverse fields, spanning medical and environmental applications and lays the groundwork for advanced health assessment and AI-based decision-making.

## Conclusions

This research showcases a biodegradable and biocompatible multifunctional nanosensor platform for cardiac monitoring that can detect various stimuli, such as chemical (VOCs), biochemical (pH and lactate), and physical (pressure) signals. The sensors exhibit superior electrical properties, rapid degradation in simulated environments, high biocompatibility, and low toxicity to heart cells as well as robust electrical performance in ex vivo experiments. Additionally, an AI prediction model was developed to demonstrate the fusion of multifunctional sensor data that can be used in real-world settings. Due to its bioresorbable and nontoxic nature, this device has the potential to be used in clinical biomedical applications without the need for complex extraction surgeries and without any risk to human health or environmental contamination.

## Experimental Section

### Materials

Polyvinylpyrrolidone (PVP), poly(vinyl alcohol) (PVA), chitosan, polyvinyl butyral (PVB), Dulbecco’s phosphate-buffered saline (DPBS), poly(3,4-ethylenedioxythiophene) polystyrenesulfonate (PEDOT:PSS), Zn NPs, TTF, LDH, Dulbecco’s modified Eagle’s medium (DMEM), trypsin, fetal bovine serum (FBS), and thiazolyl blue tetrazolium bromide were purchased from Sigma-Aldrich (St. Lous, MO). Penicillin–Streptomycin 10× was purchased from Biological Industries (Beit-Haemek, Israel). All chemical solutions were purchased from Bio-Lab Ltd. (Jerusalem, Israel), without any further purification before use. Purified water was used for the preparation of the reagents and synthesis. All solutions were prepared using Milli-Q water (18.2 MΩ cm, Millipore, Bedford, MA, USA).

### Chemical and Electrical Characterization

Keithley 2536A and Keithley 2450 Graphical SourceMeter (SMU) Instruments were used to measure the electrical behavior of the fabricated sensors. Universal Laser Systems VersaLASER (VLS) Laser cutter was used to create the sensors’ shapes and masks. Bruker (Tensor 27) FTIR equipped with a high-sensitivity LN-cooled MCT detector and attenuated total reflectance spectroscopy and FEG-equipped FEI Talos 200C high-resolution transmission electron microscope was used for the chemical characterization of the samples.

### Fabrication of Substrate Film and the Electrodes

PLA (50 mg/mL) was used for fabricating the substrate film, and the polymer was dissolved thoughtfully at room temperature. Then, the sample was poured into a glass template and left to dry overnight. An electrode mask was then applied to the dry film of PLA and magnesium (Mg) was deposited (500 nm layer) by thermal evaporation forming the electrode patterns.

### Synthesis of Silver Nanowires (Ag NWs)

2.5 gr PVP was mixed in 40 mL ethyl glycol solution and 100 μL 0.15 M FeCl_3_ in ethyl glycol at 160 °C for 5 min. 100 μL 0.15 M NaCl was added as a catalyst, followed by adding 10 mL 1.5 M AgNO_3_ dropwise while mixing until the color of the mixture changed to light silver. After 2 h, methanol was added to the mixture to stop the reaction. The sample was centrifuged over several cleaning cycles to obtain the final product of the pure Ag NWs.

### Fabrication of pH Sensor

The sensing layer was prepared by drop casting PEDOT:PSS as a protective and conductive layer. A powder of PANI solution in IPA (10 mg/mL) was prepared with 3% PVA and then spray-coated homogeneously on the PEDOT:PSS layer. For fabricating the reference electrode, Ag NWs with 3% PVA were spray-coated on the Mg reference electrode, and then Ag/AgCl paste was applied. A solution of NaCl and PVB in methanol was applied and dried for 30 min at room temperature to coat the reference electrode.

### Fabrication of Lactate Sensor

The sensing layer was prepared by drop casting PEDOT:PSS. Chitosan was dissolved and stirred for 1 h in 2% acetic acid to prepare 1% chitosan; 1 mL of this solution was mixed with 2 mg of Zn NPs followed by ultrasonication for 30 min. Lactate dehydrogenase (770U) was mixed well in a 2:1 (v/v) ratio with the Chitosan/Zn NPs solution to prepare the enzyme mixture. A mediating layer was mixed with 25 mg/mL TTF solution in acetone and was added to 1.25 mg/mL Zn NP dispersion in a ratio of 1:5 (v/v) and drop cast on the PEDOT:PSS layer. Then, the enzyme mixture (5 μL) was drop cast on the meditating layer, and the sensors were kept overnight at 4 °C until use. The reference electrode was prepared the same way as for the pH sensor.

### Fabrication of VOC Sensors

Zn NPs, 40–60 nm average particle size (10 mg/mL), were suspended in ethanol by sonicating for 20 min. 0.1% Acetic acid and 3% purified water (PW) were added and mixed with the Zn NPs thoroughly. The functional groups were added in the ratio of 1:500 with the Zn NP solution and then heated to 80 °C overnight while mixing. Samples were then centrifuged and washed for several cycles to remove the residues. The Zn-functionalized nanoparticles were dropped onto the sensing area and then heated to 60 °C under vacuum overnight, followed by UV annealing.

### VOC Sensor Experiments

The sensors were affixed to a board and placed in a stainless-steel chamber. The various VOCs comprising hexanol, hexane, *p*-xylene, hexanal, furfural, acetonitrile, hexanoic acid, and acetic acid were administered to the sensors through a computer-regulated bubbler in a continuous stream. To acquire a baseline, the sensors were subjected to N_2_ for 30 min before they were exposed to high concentrations of each of the VOCs.

### Fabrication of the PCL Membrane

PCL was dissolved in a mixture solvent of *N*,*N*-dimethylformamide and dichloromethane with a concentration of 14 wt % and stirred at 50 °C for 5 h to obtain a homogeneous solution. The PCL solution was then transferred to a plastic syringe with a 21G stainless-steel needle and the needle was connected to a positive high-voltage supply. A grounded rotating plate covered with aluminum foil was used as the collector, which was connected to a negative high-voltage supply. The distance between the needle and the collector was fixed at 16.5 cm. During electrospinning, the flow rate of the solution was controlled by a syringe pump (2 mL/h) and the rotational speed of the collector was 600 rpm under a positive voltage of 17 kV and negative of 1 kV. The average humidity and temperature were RH 50% and 22 °C. After electrospinning, the electrospun membrane was dried at room temperature in a vacuum oven overnight.

### Cell Culture

The H9C2 (2–1) cell line derived from the embryonic BD1X rat heart tissue was obtained from Prof. Shulamit Levenberg’s lab (Faculty of Biomedical Engineering, the Technion). The cells were cultured in DMED culture media supplemented with 10% FBS and 1% penicillin–streptomycin at 37 °C with incubation in the air plus 5% CO_2_.

### Cell Viability Assay

H9C2 (2–1) cells were cultured in 6-well culture plates (1 × 10^5^ cells/well) overnight. Samples were sterilized using 70% EtOH and then under UV for 15 min before being added to the well plate for 48 h and 1 week. The cells were washed with PBS, collected by adding Trypsin, and then counted for the direct number assay in a cell counting chamber using Trypan blue.

### Cytotoxicity Assays

H9C2 (2–1) cells were cultured in 96-well culture plates (1 × 10^4^ cells/well) overnight. Patch component samples were cut into 4 mm × 4 mm squares, sterilized using 70% EtOH, then placed under UV light for 15 min, and then each sample was added to the 96-well plate. Twenty microliters of MTT stock solution (5 mg/mL) was added to each culture and incubated for 3 to 4 h. At the end of the incubation, the medium was removed and the converted dye was dissolved with 150 μL of DMSO. Then, the absorbance was measured at a wavelength of 570 nm with background subtraction at 630–690 nm.

### iPSC Culture

iPSCs were generated from omental stromal cells and were a kind gift from Dr. Rivka Ofir from Ben Gurion University. The undifferentiated cells were cultivated on 10 cm culture plates precoated with Matrigel (BD, Franklin Lakes, New Jersey) diluted to 250 μg/mL in DMEM/F12 (Biological Industries). Cells were maintained in a NutriStem (Biological Industries) medium containing 0.1% Penicillin/Streptomycin (Biological Industries) and cultured under a humidified atmosphere at 37 °C with 5% CO_2_. The medium was refreshed daily, and cells were passaged at 70% confluence by treatment with 1 mL of ReLeSR (STEMCELL Technologies, Vancouver, Canada).

### CM Differentiation from iPSCs

Prior to differentiation, cells were passed to 6-well plates. NutriStem was refreshed daily until iPSCs reached 100% confluence. At that point (Day 0), the medium was changed to 3 mL of RPMI (Biological Industries), supplemented with 0.5% l-glutamine (Biological Industries), B27-Insulin (Invitrogen, Carlsbad, California), and 4.5 μM CHIR-99021 (Tocris, Bristol, UK). On Day 2, the medium was changed to 3 mL of RPMI supplemented with 0.5% l-glutamine, B27-Insulin, and 5 μM IWP-2 (Tocris). On Day 4, the medium was changed to 3 mL of RPMI supplemented with 0.5% l-glutamine and B27-Insulin, and this medium was refreshed on Day 6. On Days 8 and 10, the medium was changed to 3 mL of RPMI supplemented with 0.5% l-glutamine and B27. From Day 12, the medium was changed to M-199 (Biological Industries), supplemented with 0.1% penicillin/streptomycin, 5% fetal bovine serum (FBS, Biological Industries), 0.6 mM CuSO_4_·5H_2_O, 0.5 mM ZnSO_4_·7H_2_O, and 1.5 mM Vitamin B12 (Sigma-Aldrich). This medium was refreshed every other day.^74^

### 3D Printing of Cardiac Patches

Cells grown on Matrigel-coated plates were incubated for 10 min with TrypLE Express (Gibco, Waltham, Massachusetts). Colonies were then mechanically triturated, and the cells were centrifuged at 300*g* for 5 min. The supernatant was removed, and the omentum gel was added at a ratio of 1 mL per 50 million cells. Cells were printed using a high-precision printhead. To create the patches’ inherent vasculature, a 10% solution of ∼300 Bloom Gelatin A (Sigma-Aldrich) in the cell medium was printed in the middle layer of the patch. The gelatin ink was maintained at 37 °C before and during its extrusion. Following extrusion, printing was paused for 5 min to allow the gelatin to cool and solidify, at which point printing was resumed. Following printing, patches were placed in a humid incubator (37 °C, 5% CO_2_) for 15 min, during which time the omentum patches underwent a process of physical cross-linking. After 15 min, M-199 was added, and the patches’ medium was changed every 2–3 days.

### Statistical Analysis

Quantitative data have been expressed as mean ± standard deviation (SD). Statistical differences were assessed using One-way ANOVA analysis. *p* < 0.05 was considered statistically significant.

## Supporting Information Available

Schematic of the design and fabrication of the electrodes, linear electrochemical correlation, characterization of the fabricated Zn NPs, detailed response of the Zn NPs, microscopic images, process of degradation, gas permeability, tSNE visualization, printing of non-cellularized patch, electrochemical response of the biosensors, class distribution of the target values, correlation matrix of the training data, confusion matrix, estimated costs, summary of the used sensors’ dataset and overall dataset, “health barcode” output, status counts, fused and singular data, and different model approaches(PDF)
Degradation of the Mg electrode (MP4)
Maintaining the conductivity of the electrodes (MP4)
Attaching the biodegradable pressure sensor to the beating 3Dprinted silicone heart (MOV)
Attaching the biodegradable pressure sensor to the beating 3Dprinted silicone heart and transmitting the data by Bluetooth to the computer (MOV)
3D printing of cardiac patch with Xanthan gum (MP4)
Patch undergoing perfusion using a red dye (MOV)
3D printed beating cardiac patch (AVI)
Beating cardiac cells (MOV)
Electrochemical response of the pH biodegradable sensor (MP4)
Electrochemical response of the lactate biodegradable sensor (MP4)

## Supplementary Material

se3c01755_si_003.mp4

se3c01755_si_004.mov

se3c01755_si_005.mov

se3c01755_si_006.mp4

se3c01755_si_007.mov

se3c01755_si_008.avi

se3c01755_si_009.mov
