# Supplementary material for: Biodegradable, Biocompatible, and Implantable Multifunctional Sensing Platform for Cardiac Monitoring
Source: ACS Sens. 2024 Jan 3;9(1):126–38. doi: 10.1021/acssensors.3c01755 (PMC10825867; doi:10.1021/acssensors.3c01755)
Supplement: Supplementary file 1 — se3c01755_si_001.pdf [file se3c01755_si_001.pdf]

Supporting Information

Biodegradable, Biocompatible and Implantable Multifunctional Sensing Platform for Cardiac Monitoring

Rawan Omar [a], *Walaa Saliba* [a], *Muhammad Khatib* [a], *Youbin Zheng* [a], *Calvin Pieters* [b], *Hadas Oved* [c], *Eric Silberman* [c], *Orr Zohar* [a], *Zhipeng Hu* [a], *Viki Kloper* [a], *Yoav Y Broza* [a], *Tal Dvir* [c,d,e,f], *Alon Grinberg Dana* [b], *Yan Wang* [g], *Hossam Haick* [a,*]

[a] Department of Chemical Engineering and Russell Berrie Nanotechnology Institute, Technion-Israel Institute of Technology, Haifa 3200003, Israel

[b] Department of Chemical Engineering, Technion - Israel Institute of Technology, Haifa 320003, Israel

[c] Shmunis School of Biomedicine and Cancer Research, Faculty of Life Sciences, Tel Aviv University, Tel Aviv, Israel

[d] Department Biomedical Engineering, Faculty of Engineering, Tel Aviv University, Tel Aviv 6997801, Israel

[e] The Chaoul Center for Nanoscale Systems. Tel Aviv University Center for Nanoscience and Nanotechnology. Tel Aviv 6997801 Israel.

[f] Sagol Center for Regenerative Biotechnology Tel Aviv University Tel Aviv 6997801 Israel.

[g] Department of Chemical Engineering, Guangdong Technion-Israel Institute of Technology (GTIIT), Shantou, Guangdong 515063, China

*Corresponding author, E-mail: hhossam@technion.ac.il

Contents:

Supplementary Figures

Figure 1 | Design and fabrication of the biodegradable nanosensors. A. Schematic of the design and fabricating of the electrodes using the thermal evaporation technique. B. Schematic of the multifunctional platform which includes pressure sensors, biosensors, and VOCs sensor array. C. A microscopic image of the fabricated Mg electrode pattern. D. Photo of the transparent PLA substrate.

Supplementary Figure 2 | A. Evaporation of the Mg electrodes on PLA substrate. B. The conductivity of the fabricated array.

Supplementary Figure 3 | Linear electrochemical correlation of the fabricated pH (A) and lactate sensors (B).

Supplementary Figure 4 | Characterization of the fabricated Zn NPs for VOCs sensing. A. XPS characterizations. B. SEM images of the Zn NPs.

Supplementary Figure 5 | Detailed response of the Zn NPs functionalized with the different thiols for various gases.

Supplementary Figure 6 | The response of the biodegradable chemirsistor for different VOCs. A. Acetonitrile. B. Hexanal. C. P-xylene. D. Acetic acid. E. Hexane. F. Hexanoic acid. G. Hexanol. H. Furfural.

Supplementary Figure 7 | Microscopic images of the cells used for the *In vitro* biocompatibility evaluation of the nanosensors array (Scale bar = 100 µm).

Supplementary Figure 8 | Process of degradation of Zn NPs in SBF solution.

Supplementary Figure 9 | A. Photo of the PCL membrane. B. Microscopic image of the PCL membrane. C. Degradation of the biodegradable nanosensors after coating with PCL membrane over time.

Supplementary Figure 10 | A. Entire coating of the biodegradable sensors using PCL membrane. B. The conductivity of the sensor, connected to a LED and immersed in SBF solution over a week.

Supplementary Figure 11 | Gas permeability of the PCL membrane over time.

Supplementary Figure 12 | Design and application of the 3D printed silicone heart model. A. The 3D design of the model. B. Photo of the 3D printed silicone heart model. C. The Bluetooth IoT device for transmission of data. D. Setting of the ex-vivo experiment conducted on silicone heart model by connecting the Bluetooth chip to the sensor and transferring the data via IoT technology to the cloud. E. The transmitted Bluetooth response of the pressure sensor over the beating of the printed heart from the software.

Supplementary Figure 13 | Design of the 3D cardiac patches.

Supplementary Figure 14 | A. Printing of non-cellularized patch under perfusion with red dye. B, C. Printing of the cellularized cardiac patches in medium.

Supplementary Figure 15 | A. The printed cardiac patch, scales bar 5 mm. B. Analysis of the calculated contraction amplitude for the printed patch.

Supplementary Figure 16 | The electrochemical response of the biosensors using the 3D printed cardiac patches. A. Response of the pH sensor. B. Response of the lactate sensor.

Supplementary Figure 17 | Class distribution of the target values in training and test data using blind dataset.

Supplementary Figure 18 | Correlation matrix of the training data, where 1 shows two features that are interchangeable due to their positive correlation, whilst scores close to 0 are independent and have no display no linear relationship.

Supplementary Figure 19 | Confusion matrix of the approach Two Model Hyperparameterised. Values in the diagonal from the top left corner to the bottom right corner are correctly predicted. Values outside this diagonal are incorrect.

Supplementary Figure 20 | tSNE visualization of the training data that has been enhanced with the SMOTE method.

Supplementary Figure 21 | The relative importance of different features in the model.

Supplementary Tables 1-8

Supplementary Notes 1-3

Supplementary Equations 1-2

Captions for Supplementary Videos 1-10

Other Supplementary Materials for this manuscript include:

Supplementary Videos 1-10

Supplementary Figures

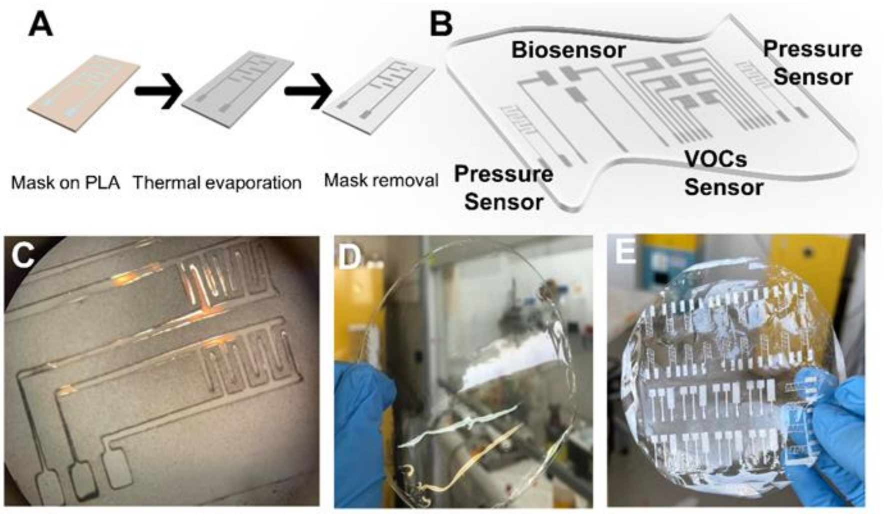

Figure S1 | Design and fabrication of the biodegradable nanosensors. A. Schematic of the design and fabricating of the electrodes using thermal evaporation technique. B. Schematic of the multifunctional platform which includes pressure sensors, biosensors, and VOCs sensor array. C. A microscopic image of the fabricated Mg electrode pattern. D. Photo of the transparent PLA substrate. E. Photo a multifunctional nanosensors array.

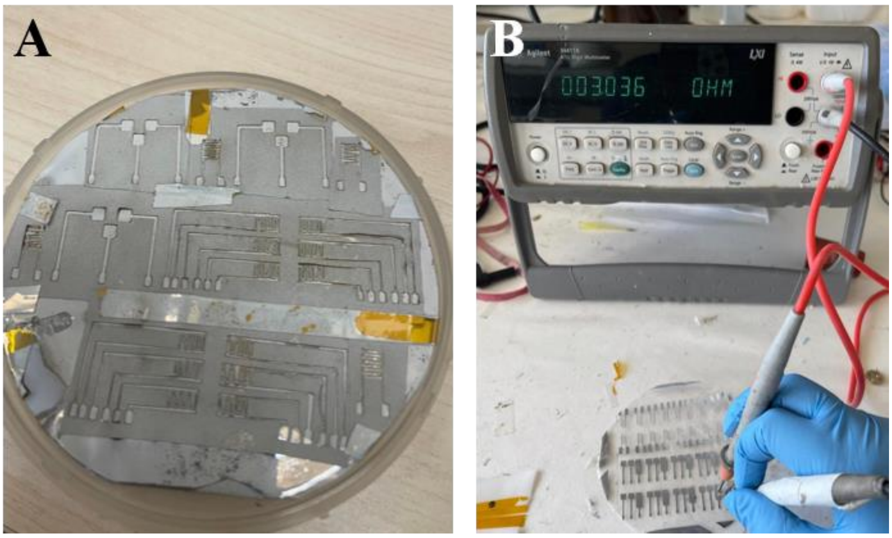

Figure S2 | A. Evaporation of the Mg electrodes on PLA substrate. B. The conductivity of the fabricated array.

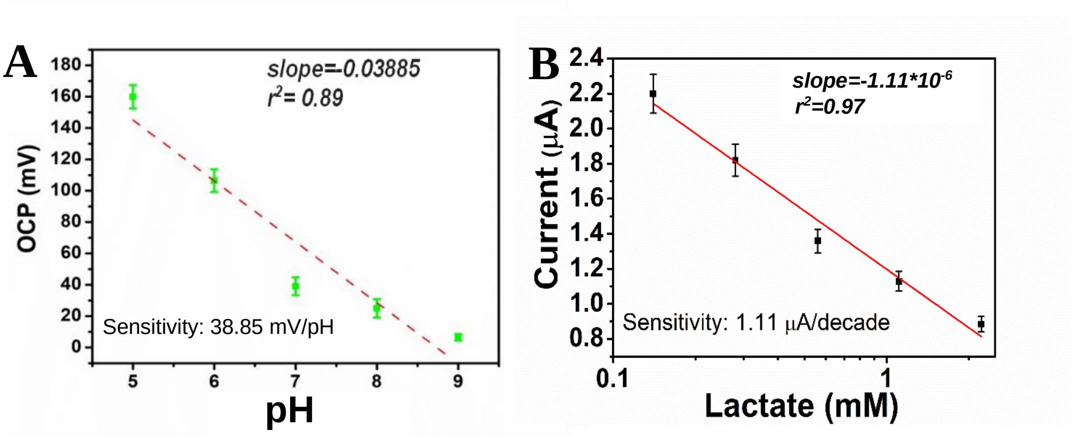

Figure S3 | Linear electrochemical correlation of the fabricated pH (A) and lactate sensors (B).

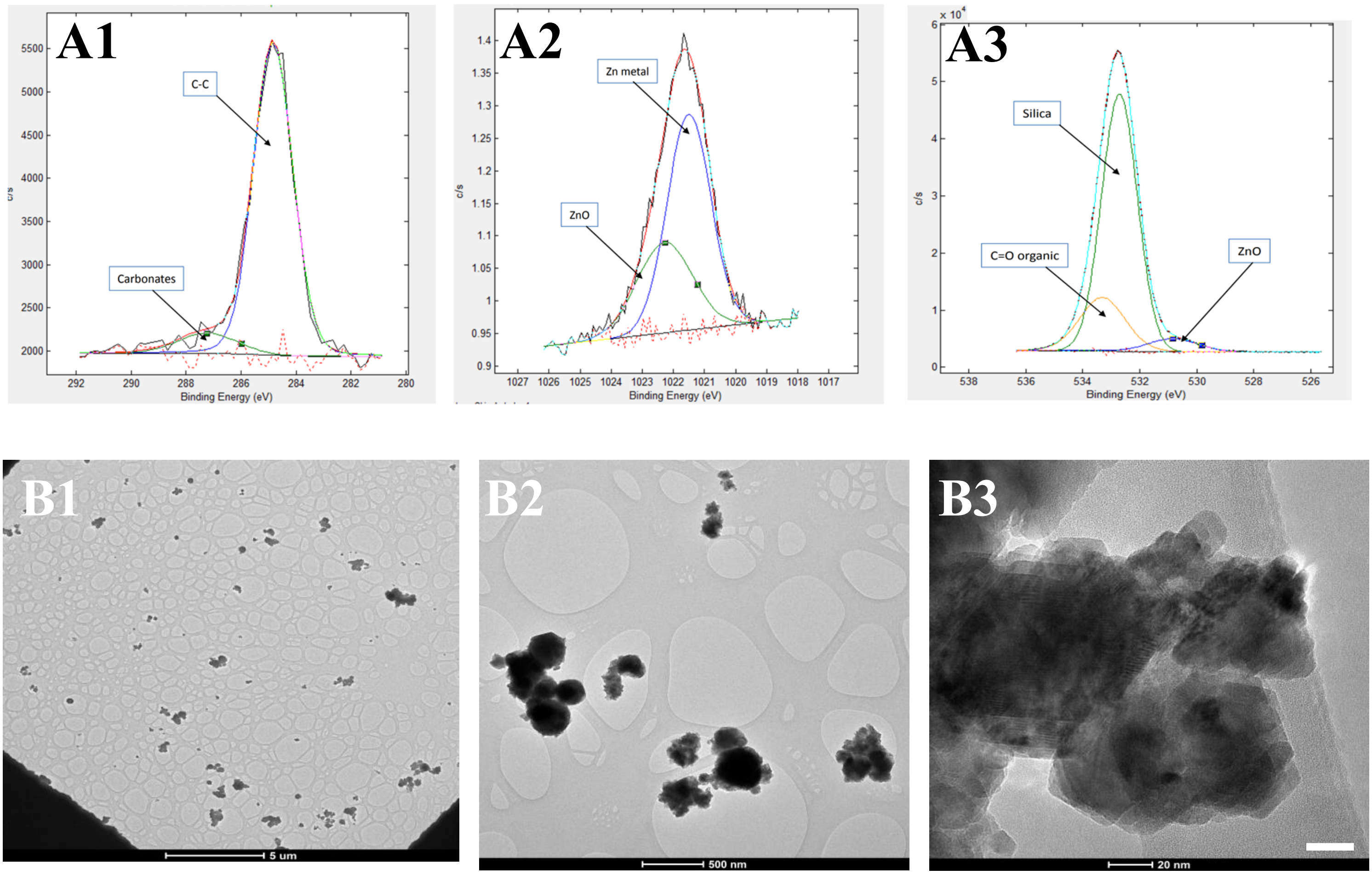

Figure S4 | Characterization of the fabricated Zn NPs for VOCs sensing. A. XPS characterizations. B. SEM images of the Zn NPs.

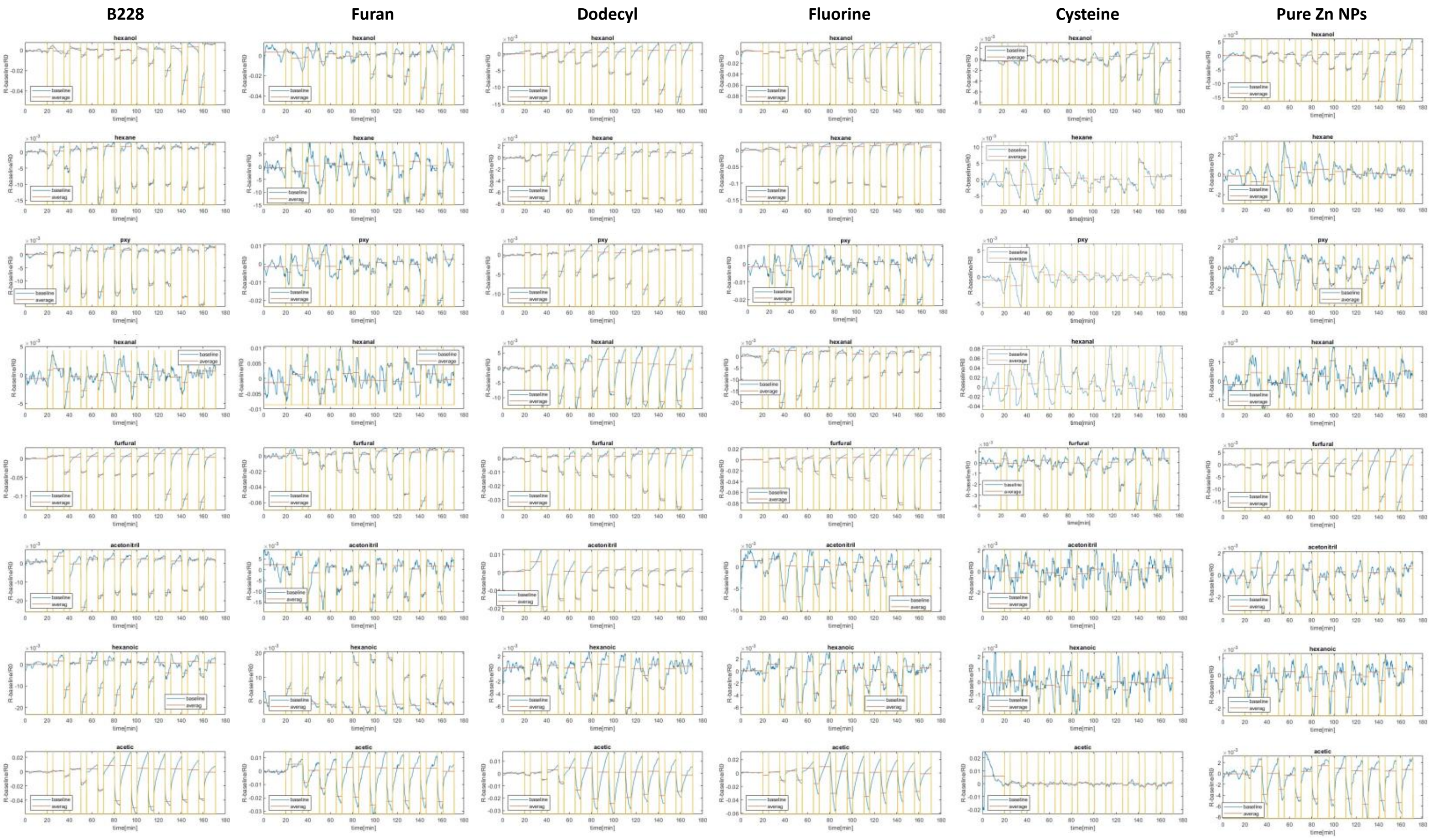

Figure S5 | Detailed response of the Zn NPs functionalized with the different thiols for various gases.

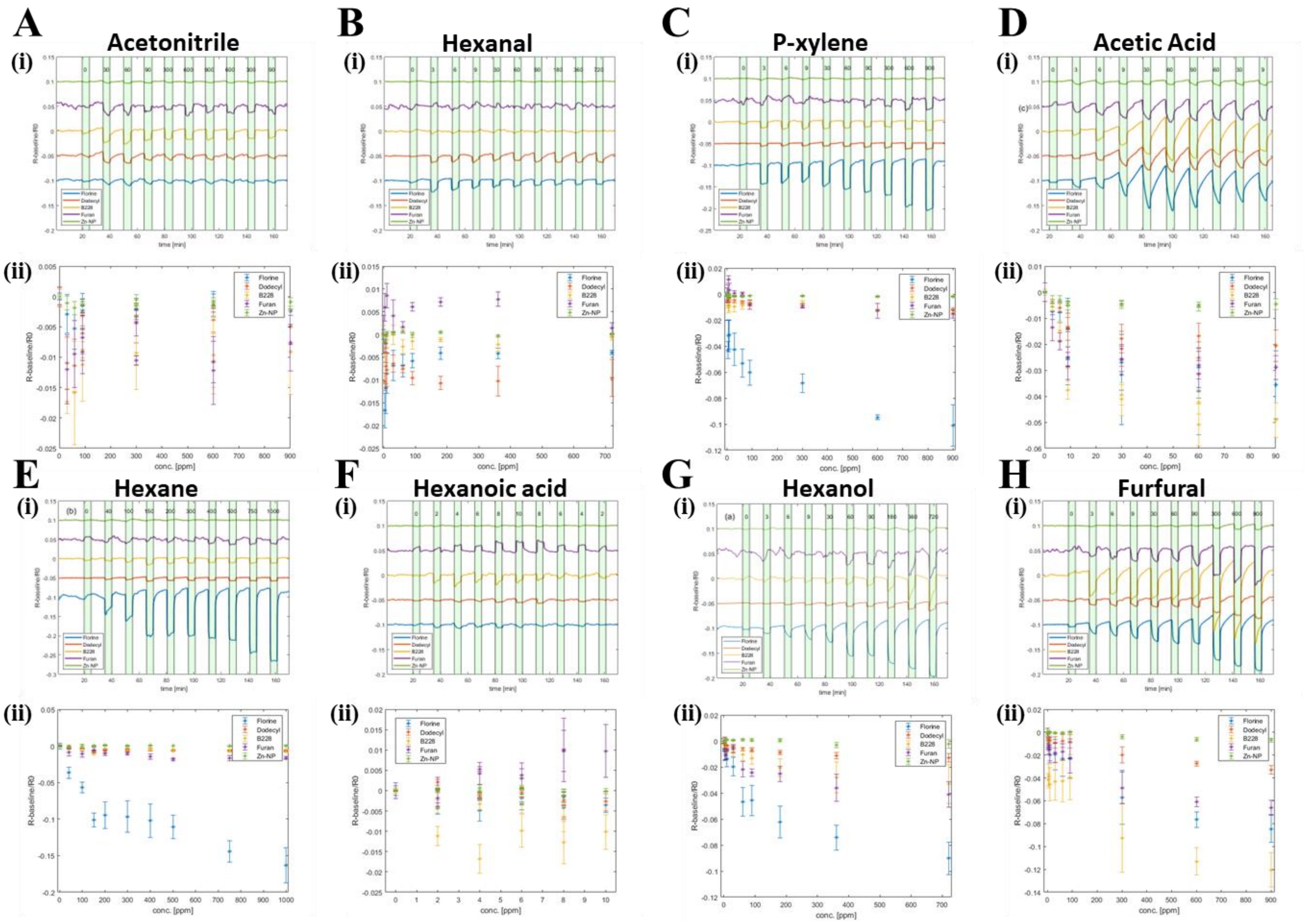

Figure S6 | The response of the biodegradable chemirsistor for different VOCs. A. Acetonitrile. B. Hexanal. C. P-xylene. D. Acetic acid. E. Hexane. F. Hexanoic acid. G. Hexanol. H. Furfural.

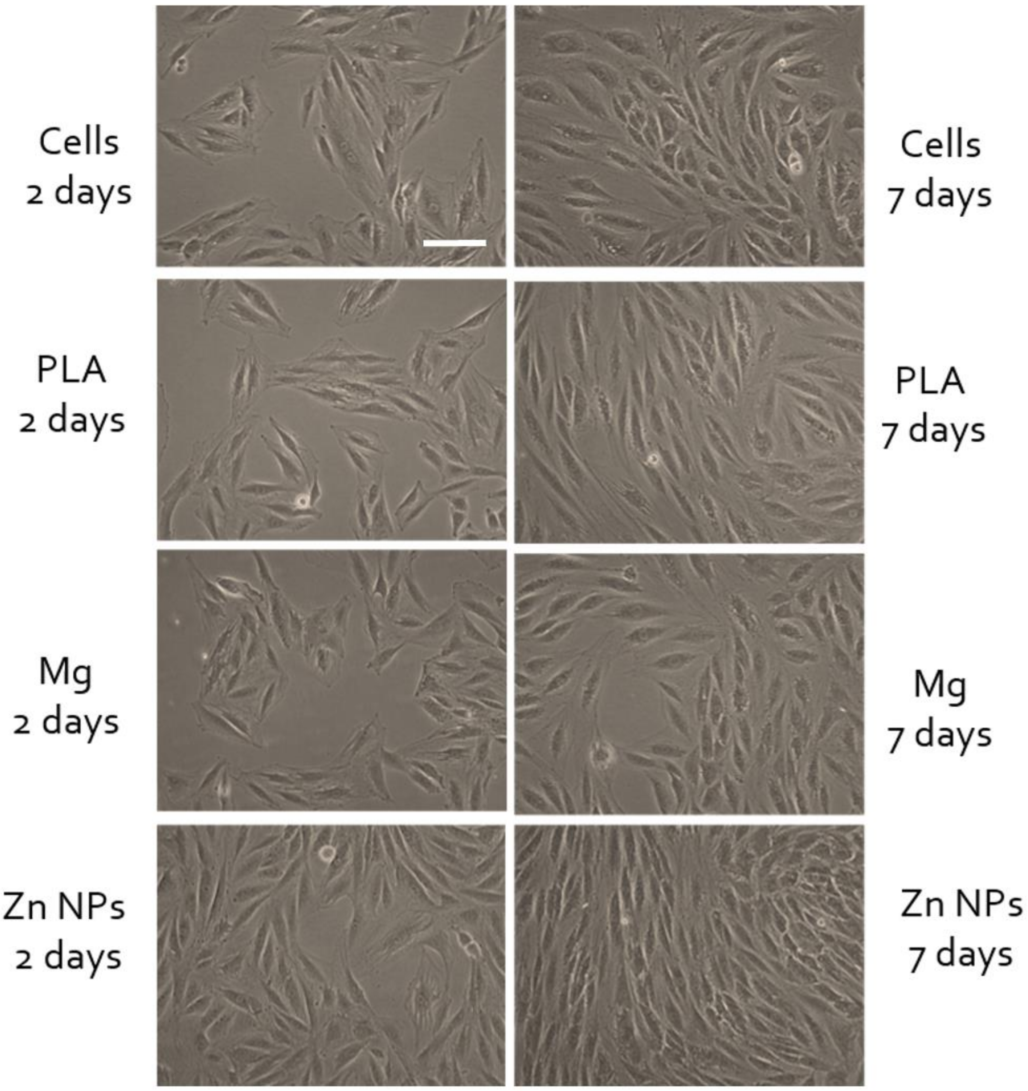

Figure S7 | Microscopic images of the cells used for the *In vitro* biocompatibility evaluation of the nanosensors array (Scale bar = 100 µm).

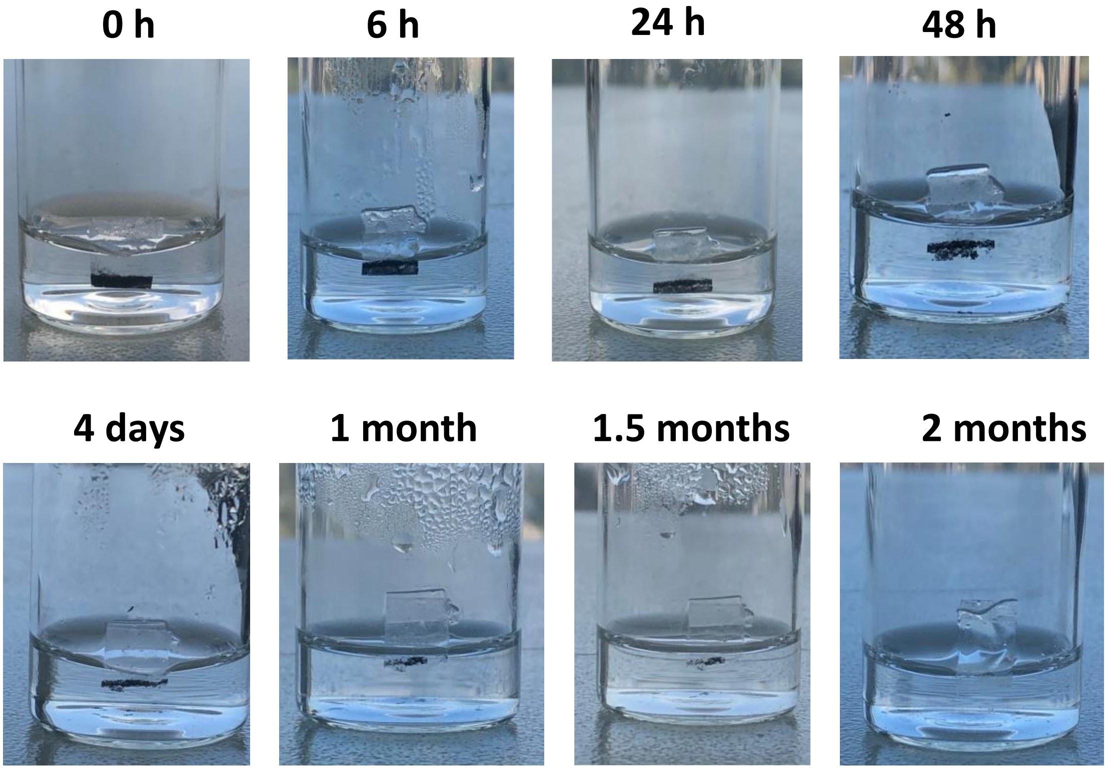

Figure S8 | Process of degradation of Zn NPs in SBF solution.

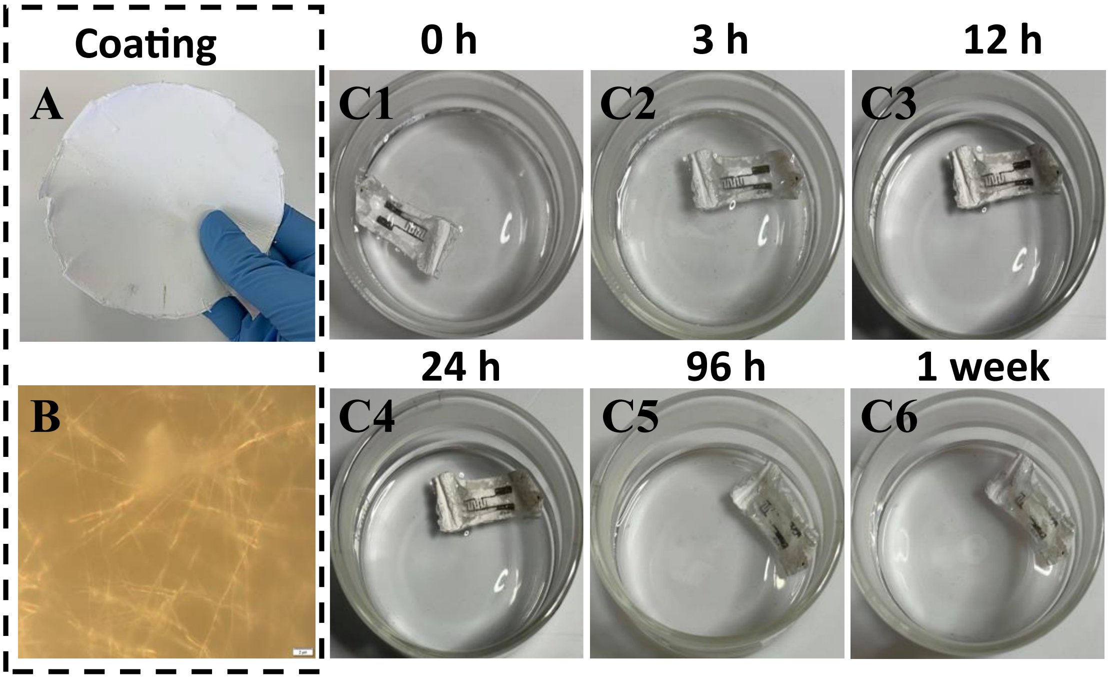

Figure S9 | A. Photo of the PCL membrane. B. Microscopic image of the PCL membrane. C. Degradation of the biodegradable nanosensors after coating with PCL membrane over time.

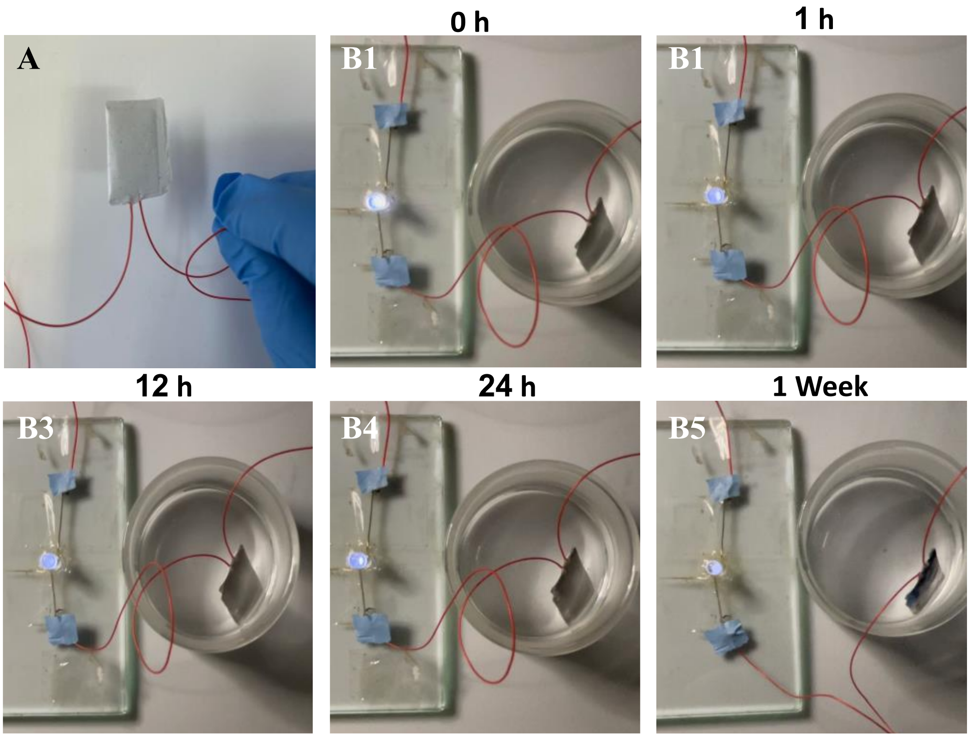

Figure S10 | A. Entire coating of the biodegradable sensors using PCL membrane. B. The conductivity of the sensor, connected to a LED and immersed in SBF solution over a week.

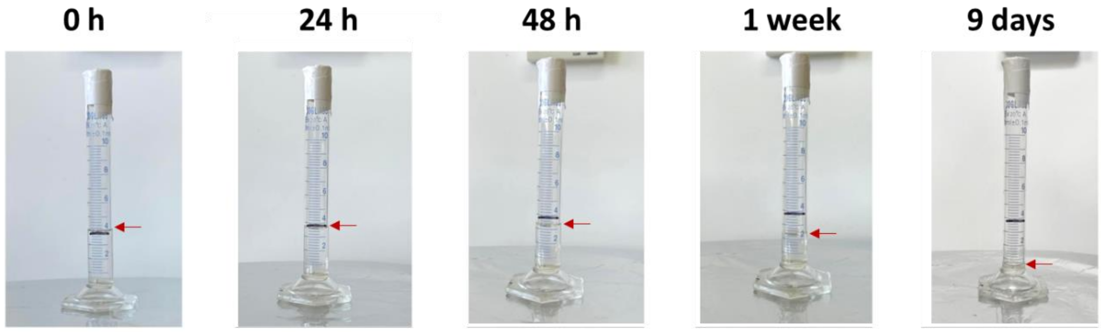

Figure S11 | Gas permeability of the PCL membrane over time.

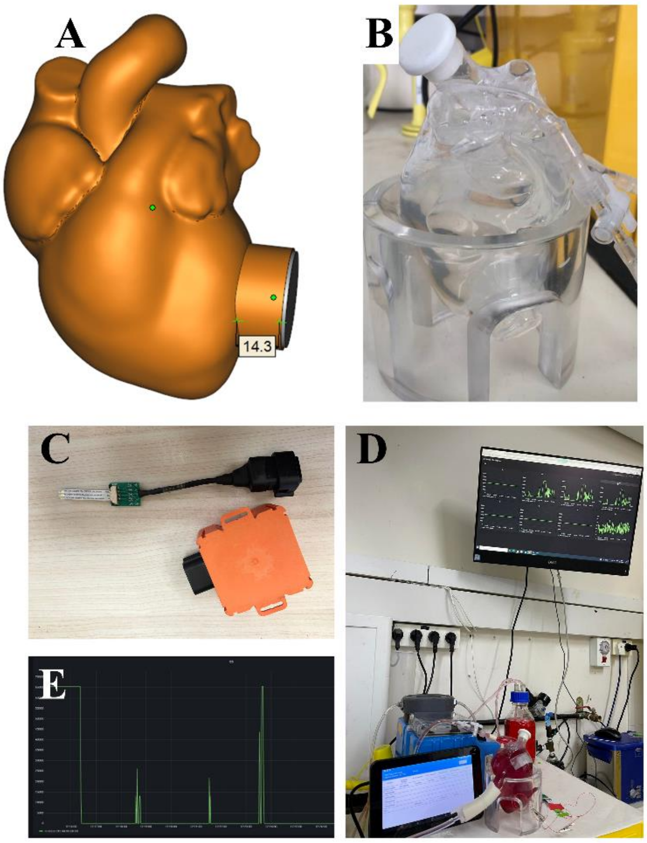

Figure S12 | Design and application of the 3D printed silicone heart model. A. The 3D design of the model. B. Photo of the 3D printed silicone heart model. C. The Bluetooth IoT device for transmission of data. D. Setting of the ex-vivo experiment conducted on silicone heart model by connecting the Bluetooth chip to the sensor and transferring the data via IoT technology to the cloud. E. The transmitted Bluetooth response of the pressure sensor over the beating of the printed heart from the software.

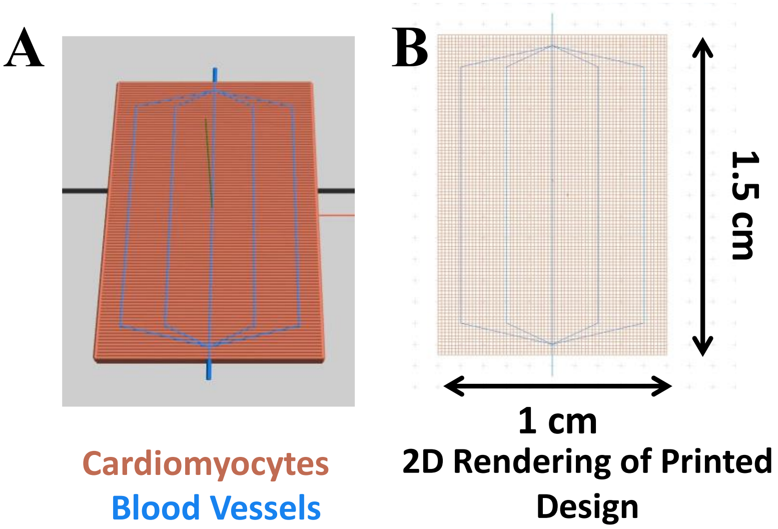

Figure S13 | Design of the 3D cardiac patches.

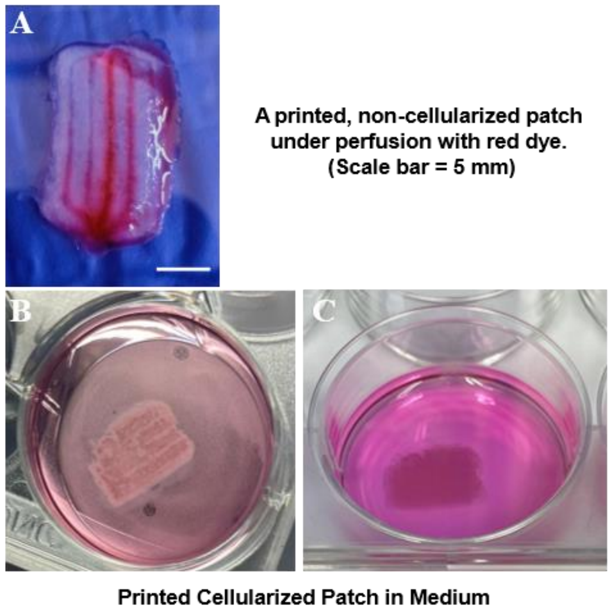

Figure S14 | A. Printing of non-cellularized patch under perfusion with red dye. B, C. Printing of the cellularized cardiac patches in medium.

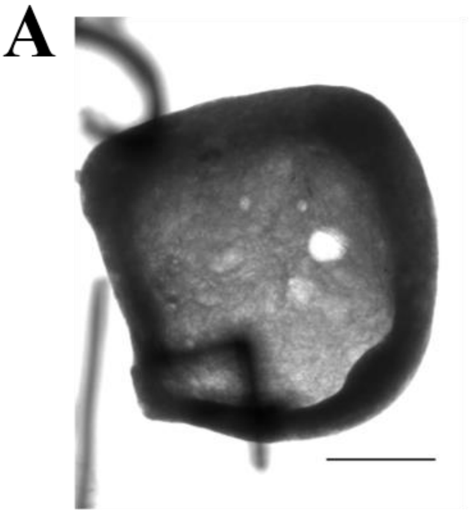

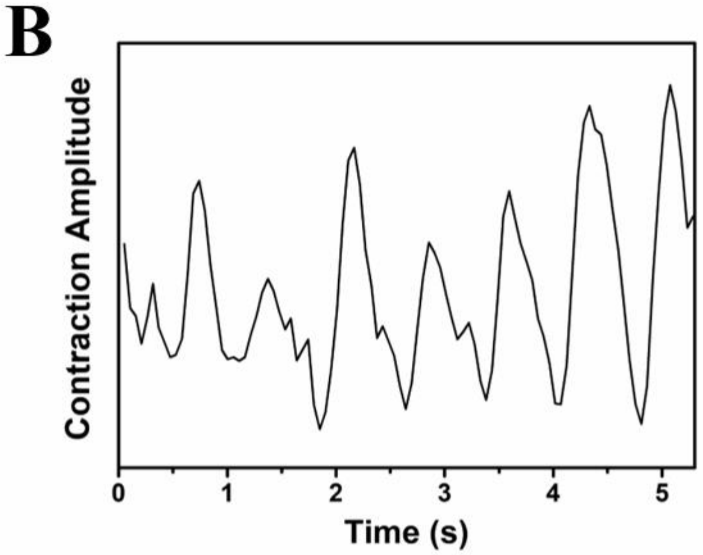

Figure S15 | A. The printed cardiac patch, scale bar 5 mm. B. Analysis of the calculated contraction amplitude for the printed patch.

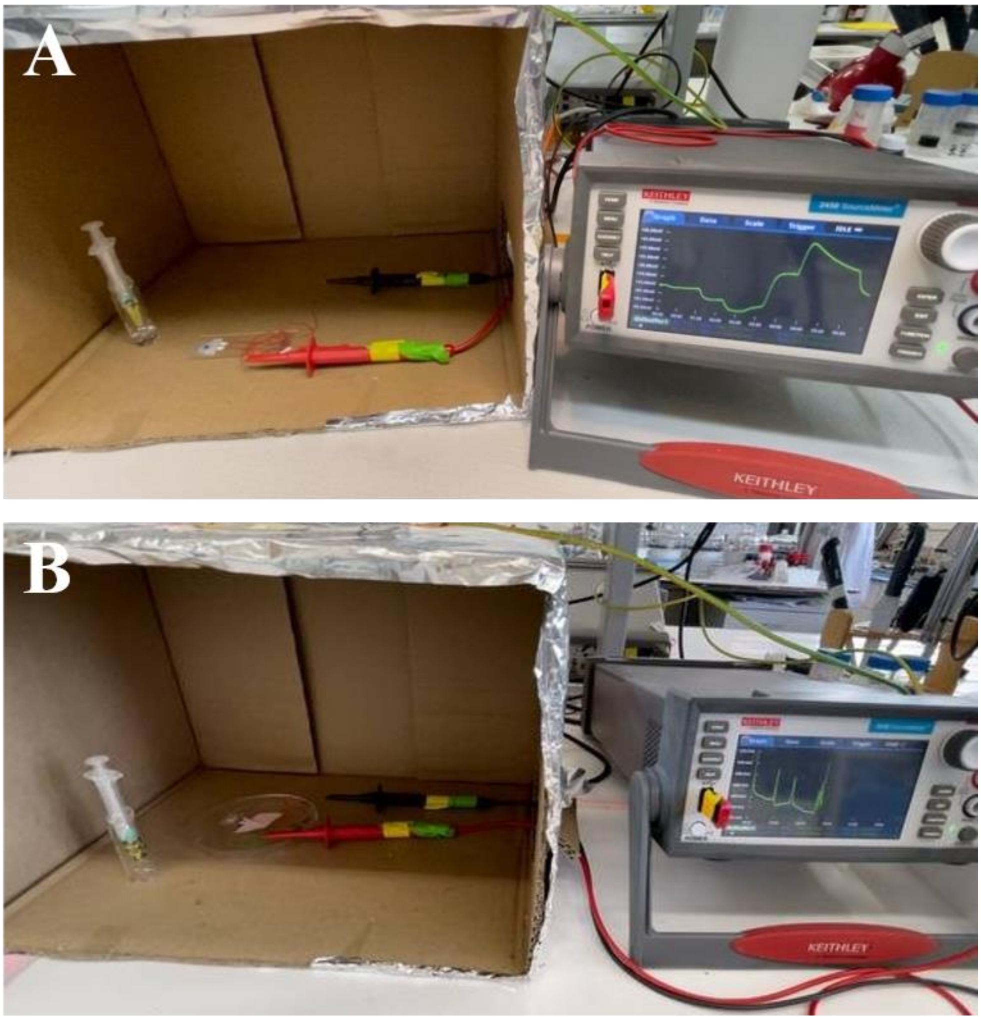

Figure S16 | The electrochemical response of the biosensors using the 3D printed cardiac patches. A. Response of the pH sensor. B. Response of the lactate sensor.

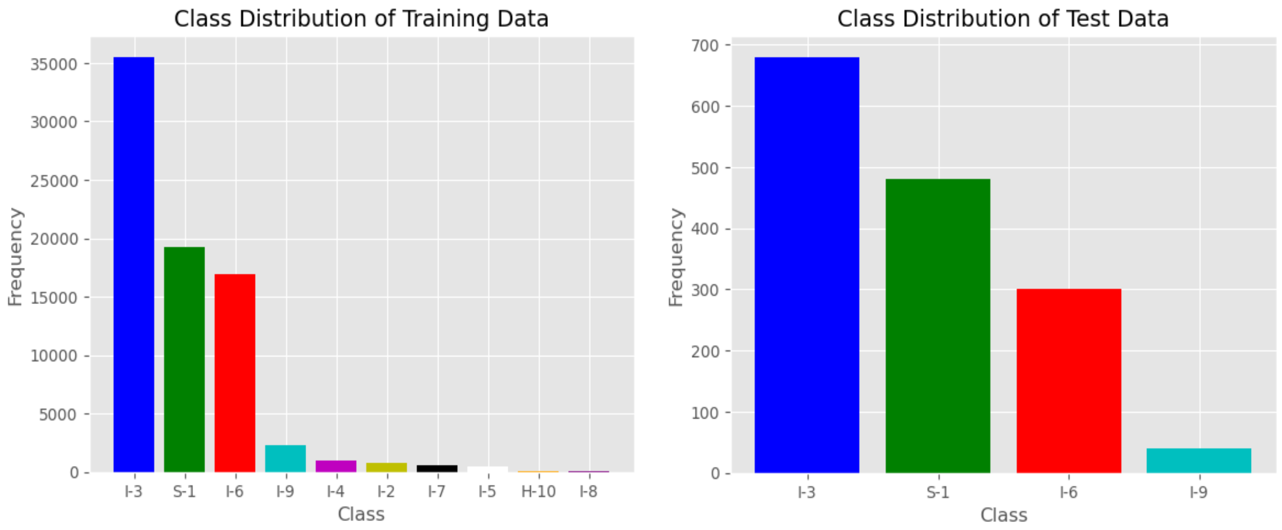

Figure S17 | Class distribution of the target values in training and test data using blind dataset.

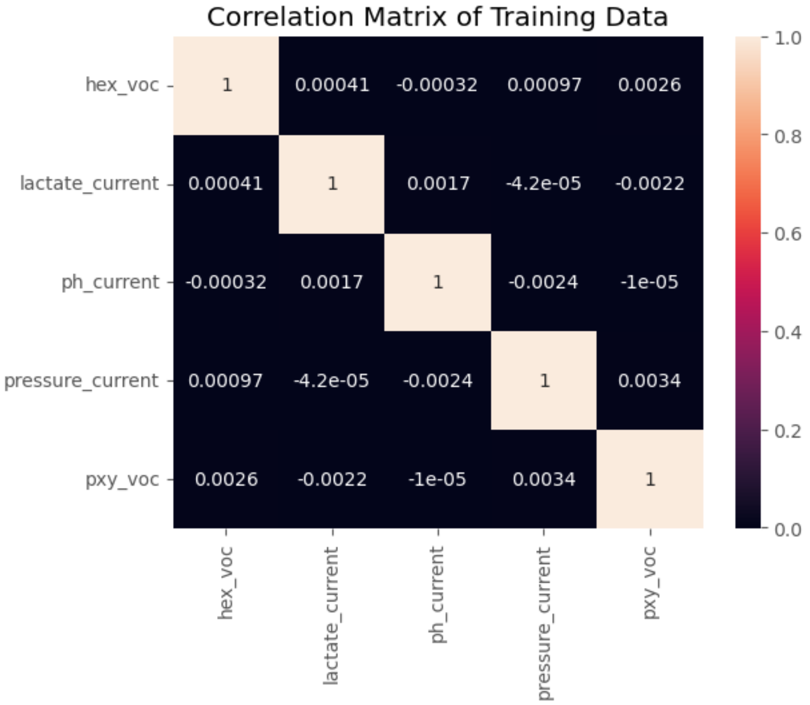

Figure S18 | Correlation matrix of the training data, where 1 shows two features that are interchangeable due to their positive correlation, whilst scores close to 0 are independent and have no display no linear relationship.

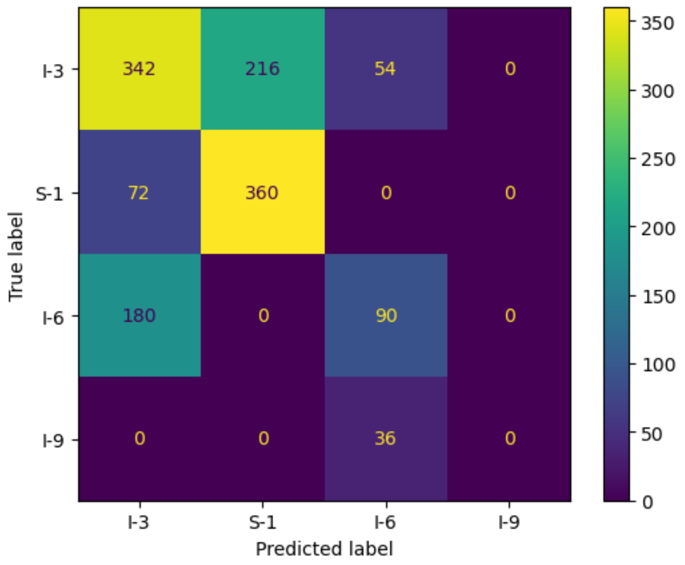

Figure S19 | Confusion matrix of the approach Two Model Hyperparameterised. Values in the diagonal from the top left corner to the bottom right corner are correctly predicted. Values outside this diagonal are incorrect.

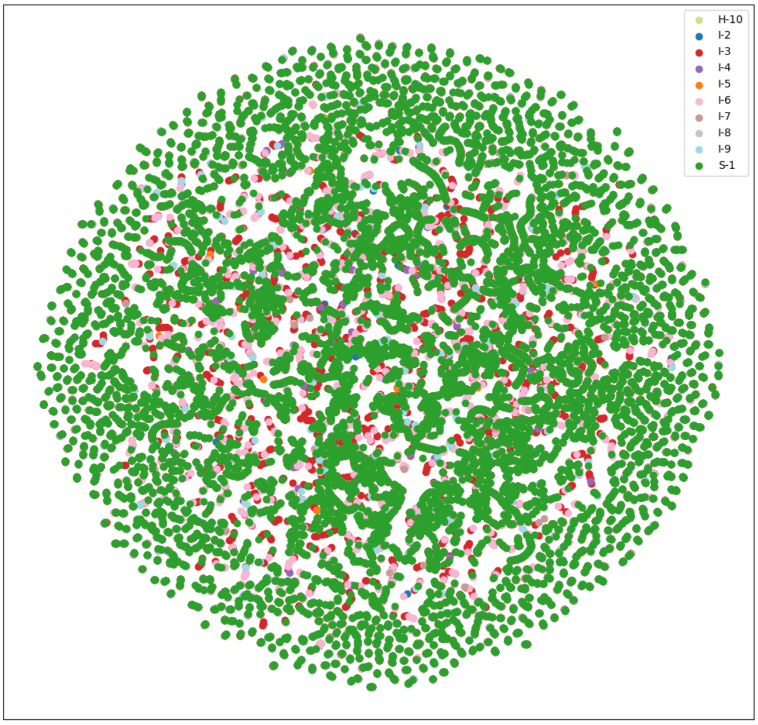

Figure S20 | tSNE visualization of the training data that has been enhanced with the SMOTE method.

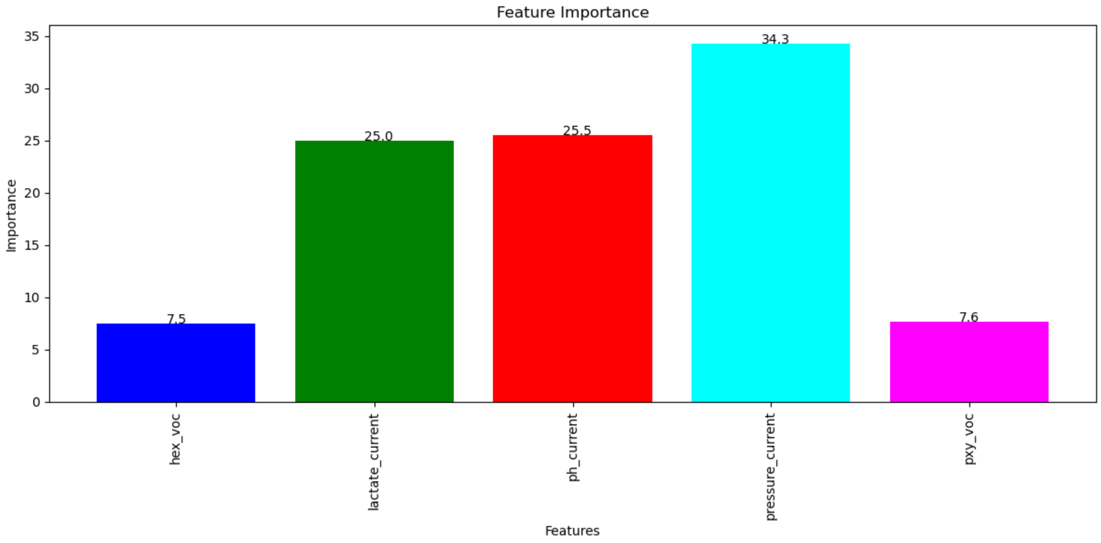

Figure S21 | The relative importance of different features in the model.

Table S1 Estimated costs of our device in comparison to current solutions in the market

Device name	Costs	Reference
Multifunctional Sensing Platform	Procedures in procedure room: $100<br>Cost of device: $35<br>Removal of device: $0 *	This work
Reveal XT	Procedures in catheterization laboratory: $836<br>Cost of device: $2,234<br>Removal of device: $609	[1,2]
Reveal LINQ	Procedures in procedure room: $119<br>Removal of device: $295	[1,2]
BioMonitor 2-AF™	Procedure supply costs: $130<br>Cost of device: $1,278<br>Removal of device: $295	[2,3]
Confirm Rx™	Cost of device: $1,985<br>Removal of device: $295	[2]

* No need for surgery for removal since the device is biodegradable.

Table S2 Summary of the used sensors' datasets

Biomarker name	Column headings	Number of 'Sick' Status	Number of 'Healthy' Status
pH	OCP(V)	8	2
Lactate	Current (A)	5	2
Pressure	R/R0	5	6
VOC: Hexane	dR dodecyl S23[Ohm], dR b228 S24 [Ohm], dR ZnPS S35 [Ohm], dR fluorine S38 [Ohm], dR Furan S32 [Ohm], dR cysteine S28 [Ohm]	8	2
VOC: p-xylene	dR dodecyl S23[Ohm], dR b228 S24 [Ohm], dR ZnPS S35 [Ohm], dR fluorine S38 [Ohm], dR Furan S32 [Ohm], dR cysteine S28 [Ohm]	8	2

Table S3 Summary of the overall dataset

	**hex_voc**	**hex_status**	**lactate_current**	**lactate_status**	**pressure_current**	**pressure_status**	**pxy_voc**	**pxy_status**	**ph_current**	**ph_status**
**count**	77000	77000	77000	77000	77000	77000	77000	77000	77000	77000
unique	*NaN*	2	*NaN*	2	*NaN*	2	*NaN*	2	*NaN*	2
top	*NaN*	sick	*NaN*	sick	*NaN*	healthy	*NaN*	sick	*NaN*	sick
**freq**	*NaN*	61600	*NaN*	55000	*NaN*	42000	*NaN*	61600	*NaN*	61600
mean	96266.9	*NaN*	1.74E-06	*NaN*	0.566725	*NaN*	121078.5	*NaN*	0.068462	*NaN*
**std**	22278.44	*NaN*	1.25E-06	*NaN*	1.044507	*NaN*	35557.15	*NaN*	0.046157	*NaN*
**min**	49998.58	*NaN*	3.50E-07	*NaN*	0.070701	*NaN*	68181.18	*NaN*	0.006453	*NaN*
**25%**	92685.12	*NaN*	6.24E-07	*NaN*	0.131496	*NaN*	87957.95	*NaN*	0.025267	*NaN*
**50%**	101892.5	*NaN*	1.39E-06	*NaN*	0.241963	*NaN*	123506.7	*NaN*	0.0609	*NaN*
**75%**	111662.6	*NaN*	2.43E-06	*NaN*	0.314923	*NaN*	156326.9	*NaN*	0.103759	*NaN*
**max**	125023.2	*NaN*	4.38E-06	*NaN*	3.838953	*NaN*	172357.7	*NaN*	0.159931	*NaN*

Table S4 The "health barcode" output for categorizing the fused sensors output. H = Healthy, S = Sick, I = Intermediate. 10- Healthy, 9-2 Intermediate, 1-Sick

pH	Lactate	Pressure	VOCs	Score
H	H	H	H	H-10
H	H	H	S	I-9
H	H	S	H	I-8
H	S	H	H	I-7
S	H	H	H	I-7
H	H	S	S	I-6
H	S	H	S	I-6
S	H	H	S	I-6
H	S	S	H	I-5
S	H	S	H	I-5
S	S	H	H	I-4
H	S	S	S	I-3
S	H	S	S	I-3
S	S	H	S	I-3
S	S	S	H	I-2
S	S	S	S	S-1

Table S5 Status counts

Status	Counts
I-3	35520
S-1	19200
I-6	16896
I-9	2304
I-4	960
I-2	800
I-7	624
I-5	520
H-10	96
I-8	80

I-3	35520
S-1	19200
I-6	16896
I-9	2304
I-4	960
I-2	800

Table S6 Fused data of all sensors when pH is considered 'Healthy'

**Status**	**Counts**
I-6	7680
I-3	4800
I-9	2304
I-7	240
I-5	200
H-10	96
I-8	80

Table S7 Singular data of only pH and while it's considered 'Healthy'

**pH_status**	**Status**
count	15400
unique	1
top	healthy
freq.	15400

In order to calculate the accuracy of the singular pH sensor compared to the fused sensor data: (96/15400)*100 = 0.62% is the accuracy when using pH sensor as a sole health indicator. 96 is the overall status for pH when it's healthy (H-10) (Table S6), and 15400 is the overall status for pH as a singular data (Table S7).

Table S8 Different model approaches, their training accuracy, and their scores when tested against the test data

Approach	Class Weight Balancing	SMOTE	Training Accuracy	F1 Score	Accuracy
1	Yes	No	1.00	0.055	0.180
2	No	Yes	0.98	0.171	0.45
3	Yes	Yes	0.88	0.148	0.365

Supplemental Note 1 Dataset creation for the Artificial Intelligence (AI)-based model

Five independent experimental datasets were used to create the AI model, including pH, lactate, pressure and two types of representative VOCs (Hexane and p-xylene), as presented in Table S2. The model was designed to classify and fuse the data to two different categories of 'healthy' and 'sick'. A larger overall dataset was created through combinations of each dataset fused with each other. The resulting table, as summarized in Table S3, contained 77000 datapoints, which can be described as a form of synthetic data in the field of Machine Learning. The data of each sensor was classified based on the physiological values of each biomarker and divided into 'H' (Healthy) and 'S' (Sick) categories. Regarding the VOCs sensors and since the array includes 6 different sensors, it was determined that when the overall measurements of the VOCs showed healthy, then the patient was healthy. If one showed healthy and the other showed sick, then the VOC classification was determined to be sick. Then, a "health barcode" was created that defines the rules of the final output, which is a score from 1 to 10, when 10 is the healthiest and 1 is the sickest status (Table S4). Lastly, Table S5 presents the value counts for the overall status classification.

Supplemental Note 2 Data preprocessing and model development

The first step in data preprocessing involved the removal of individual statuses of each sensor in the overall dataset so that the target column, 'status', remained. Thus, the resulting table contained the headings:

- 'hex_voc' (**Feature Column**)

- ‘lactate_current’ (**Feature Column**)
- ‘ph_current’ (**Feature Column**)
- ‘pressure_current’ (**Feature Column**)
- ‘pxy_voc’ (**Feature Column**)
- ‘status’ (**Target Column**)

The next step required feature scaling, as the features within the dataset were not of the same scale. Feature scaling was performed using a Min Max Scaler. The following formulation is shown in Equation S1.

Equation S1 Min-Max scaling equation

For each feature j (1 <= j <= n):

X_scaled[:, j] = (X[:, j] - X[:, j].min()) / (X[:, j].max() - X[:, j].min()) * (max - min) + min

Supplemental Note 3 Model validation using a blind dataset

The initial training data comprised independent features that had no interdependencies. For instance, a patient's 'Lactate Current' readings bore no correlation to 'pH current' values. To construct a coherent data-frame, these independent features underwent a cross-merging process. Subsequently, a rule-based system was employed to deduce the target value. For example, if Feature 1 and Feature 2 were indicative of 'Sick' while Feature 3 signaled 'Healthy,' the resultant target value would be 'Sick.' This rule application, however, represents a simplified version of our dataset's actual construction, which adhered to a detailed status rules table to determine a data point's overall status, classifying it into one of several categories: H-10, I-9, I-8, I-7, I-6, I-5, I-4, I-3, I-2, S-1. Similar pre-processing was required for the test dataset to align it with the training dataset's structure. The class distribution visualization (Figure S17) revealed significant disparities in the data points for each target value within the training data, hinting at a potential bias toward more prevalent target values during the training phase.

Further, a correlation matrix (Figure S18) underscored the absence of relationships between features, advocating for the adoption of complex machine learning models

rather than linear ones. The CatBoost Classifier, renowned for its adeptness at handling categorical features, was selected for its speed and suitability for large datasets. Three distinct training methodologies were experimented incorporating class weight balancing, Synthetic Minority Oversampling Technique (SMOTE), or a combination of both (Table S8). The class weight balancing method was an inverse class frequency weighting, as described in Equation S2.

Equation S2 Inverse class frequency weighting

$$w_i = \frac{N}{C \times n_i}$$

Given:

- N = Total number of samples in the training dataset.
- C = Total number of unique classes.
- n_i = Number of samples of class i.
- w_i = Weight for class i.

The model's evaluation of the test data involved various methods, including F1 Scoring, a confusion matrix, and accuracy. Despite the perfect training accuracy demonstrated by Approach 1, its low F1 Score and overall accuracy pointed to significant overfitting. Approach 2 yielded a better balance between training accuracy and generalization, suggesting a more promising avenue for hyperparameter tuning. Approach 3, while presenting a reasonable balance, hinted at potential underfitting. Thus, Approach 2 was chosen for hyperparameter optimization (Table S8). Post-hyper parameterization, the Approach 2 model exhibited a modest improvement in F1 score and accuracy (0.200 and 0.528, respectively), as depicted in the confusion matrix (Figure S19). Notably, the model predicted the highest number of 'True Label' for 'S-1' but showed confusion between 'I-3' and 'I-6' and failed to predict 'I-9' correctly. Further scrutiny of the training data revealed homogeneity and label overlap, as indicated by the tSNE visualization (Figure S20), enhanced by SMOTE. This overlap poses a challenge to the model's

differentiation capabilities. An analysis of feature importance, Figure S21, showed three features significantly influencing the model's predictive capacity. However, 'hex_voc' and 'pxy_voc' were identified as having minimal importance. It is crucial to approach the interpretation of these feature importance metrics with circumspection. These metrics shed light on the relative influence each predictor wields over the model's decisions, yet they should not be regarded as definitive due to the model's suboptimal performance. The model's suboptimal performance is primarily reflected in its inability to generalize beyond the training dataset, as evidenced by the disparity between the training accuracy and validation metrics like the F1 Score.

Other supplementary materials for this manuscript include:

Supplementary Videos 1-10

Captions for Supplementary Videos 1-10:

Supplementary Video 1 | Degradation of the Mg electrode on PLA substrate in SBF solution over time.

Supplementary Video 2 | Maintaining the conductivity of the electrodes by lowering the degradation rate in SBF solution by coating the with PCL membrane.

Supplementary Video 3 | Attaching the biodegradable pressure sensor to the beating 3D printed silicone heart.

Supplementary Video 4 | Attaching the biodegradable pressure sensor to the beating 3D printed silicone heart and transmitting the data by Bluetooth to the computer.

Supplementary Video 5 | 3D printing of cardiac patch with Xanthan gum (1.11% in DDW) with colored micro-polystyrene beads to show the distinct bio-inks used in the printing.

Supplementary Video 6 | Patch undergoing perfusion using a red dye.

Supplementary Video 7 | The 3D printed beating cardiac patch, scale bar=5 mm.

Supplementary Video 8 | Beating cardiac cells incorporated in the printed patch.

Supplementary Video 9 | The electrochemical response of the pH biodegradable sensor within the 3D printed cardiac patch.

Supplementary Video 10 | The electrochemical response of the lactate biodegradable sensor within the 3D printed cardiac patch.

Supplementary references:

(1) Kanters, T. A.; Wolff, C.; Boyson, D.; Kouakam, C.; Dinh, T.; Hakkaart, L.; Rutten-Van Mölken, M. P. M. H. Cost Comparison of Two Implantable Cardiac Monitors in Two Different Settings: Reveal XT in a Catheterization Laboratory vs. Reveal LINQ in a Procedure Room. *Europace* **2016**, *18* (6), 919–924. https://doi.org/10.1093/EUROPACE/EUV217.

(2) Edwards, S. J.; Wakefield, V.; Jhita, T.; Kew, K.; Cain, P.; Marceniuk, G. Implantable Cardiac Monitors to Detect Atrial Fibrillation after Cryptogenic Stroke: A Systematic Review and Economic Evaluation. *Health Technol Assess* **2020**, *24* (5), 1. https://doi.org/10.3310/HTA24050.

(3) Awad, K.; Weiss, R.; Yunus, A.; Bittrick, J. M.; Nekkanti, R.; Houmsse, M.; Okabe, T.; Adamson, T.; Miller, C.; Alawwa, A. K. BioMonitor 2 In-Office Setting Insertion Safety and Feasibility Evaluation with Device Functionality Assessment: Results from the Prospective Cohort BioInsight Study. *BMC Cardiovasc Disord* **2020**, *20* (1). https://doi.org/10.1186/S12872-020-01439-8.
